# The role of an ultrasound-responsive injectable piezoelectric hydrogel in promoting nerve regeneration and alleviating neuropathic pain

**DOI:** 10.7150/thno.131276

**Published:** 2026-06-25

**Authors:** Zhaoyang Guo, Weiqian Jiang, Wentao Zhang, Zhongju Liu, Hang Zhou, Hang Liu, Lin Wang, Jiahao Zhang, Xinliang Peng, Xingyu Yang, Maohui Li, Hanchao Liang, Zhongyuan He, Rui Deng, Yongjun Dang, Wei Fu, Keyu Wei, Chao Xie, Zhong-Liang Deng, Youliang Ren, Lei Chu

**Affiliations:** 1 Department of Orthopaedics, The Second Affiliated Hospital of Chongqing Medical University, Chongqing 400010, China.; 2 Department of Orthopaedics, Guizhou Provincial People's Hospital, Guiyang 550000, China.; 3 Department of Plastic and Maxillofacial Surgery, The Second Affiliated Hospital of Chongqing Medical University, Chongqing 400010, China.; 4 Center for Musculoskeletal Research, University of Rochester Medical Center, Rochester, NY 14642, USA.; 5 Novel Target and Therapeutic Intervention Laboratory, The Second Affiliated Hospital of Chongqing Medical University, Chongqing 400010, China.; 6 National Engineering Research Center for Tissue Restoration and Reconstruction South China University of Technology, Guangzhou, China.; 7 Health Science Center, Xi’an Jiaotong University, 76 Yanta West Road, Xi’an 710061, China.; 8 Department of Pain Management, The Second Affiliated Hospital of Chongqing Medical University, Chongqing 400010, China.

**Keywords:** neuropathic pain, piezoelectric hydrogel, ultrasound-responsive biomaterials, peripheral nerve injury, neural regeneration

## Abstract

**Background:**

Neuropathic pain (NP) resulting from peripheral nerve injury (PNI) represents a major clinical challenge. Although electrical stimulation (ES) has demonstrated therapeutic benefits, its clinical translation is limited by the invasiveness of electrode implantation, which often requires large surgical incisions and percutaneous wiring, increasing tissue damage and infection risk.

**Methods:**

This study developed an ultrasound-responsive, injectable, and biodegradable piezoelectric hydrogel (sPLLA-Gel) composed of electrospun poly-L-lactic acid (PLLA) and methacryloyl gelatin (GelMA). The physicochemical characteristics, biocompatibility, and piezoelectric performance were systematically evaluated. *In vitro*, ultrasound-activated sPLLA-Gel was examined for its ability to promote neural stem cell (NSC) differentiation and regulate macrophage polarization. *In vivo*, the hydrogel was injected into the sciatic nerve injury site of a chronic constriction injury (CCI) rat model, followed by behavioral, electrophysiological, histological, and transcriptomic analyses.

**Results:**

The sPLLA-Gel hydrogel exhibited excellent injectability, biodegradability, and ultrasound-induced electrical responsiveness. *In vitro* studies confirmed its biocompatibility and demonstrated that ultrasound-activated sPLLA-Gel promoted neural stem cell differentiation, axon-like neurite outgrowth, and favorable macrophage polarization. *In vivo*, the hydrogel was injected into the injury site of a chronic constriction injury rat model. Behavioral and electrophysiological analyses revealed that ultrasound-stimulated sPLLA-Gel enhanced myelin and axon regeneration, improved motor function, and alleviated NP. These therapeutic effects correlated with reduced spinal glial cell activation and decreased pro-inflammatory cytokine expression. Transcriptomic analysis and further validation suggested that sPLLA-Gel’s analgesic and neuroprotective effects may arise from the inhibition of TRPV1 expression and the NF-κB signaling pathway.

**Conclusions:**

This study demonstrates the potential of this ultrasound-responsive piezoelectric hydrogel to promote peripheral nerve regeneration and mitigate NP, offering a promising minimally invasive strategy for treating sciatic nerve injury-related NP.

## Introduction

Neuropathic pain (NP) is a group of chronic disabling diseases caused by a lesion or disease of the somatosensory nervous system^1^-^3^, affecting approximately 6.9% – 10% of the global population^4^, and severely impairing patients' quality of life^5^. Among them, peripheral NP is the most common type^6^-^8^, usually caused by peripheral nerve injury (PNI)^6^,^9^ resulting from trauma^10^ or surgical procedures^11^-^14^. PNI induces spinal glial cell activation, triggering an inflammatory cascade characterized by excessive release of cytokines and chemokines. This neuroinflammatory response drives central sensitization, contributing to NP development and persistence^15^.

Currently, pharmacological therapy remains the mainstay of NP management, with commonly used drugs including antidepressants, nonsteroidal anti-inflammatory drugs (NSAIDs), and opioids^16^. Although the above treatments can relieve symptoms to some extent, their efficacy is often limited and they are often accompanied by significant adverse reactions. Therefore, developing alternative treatment strategies has become an urgent problem to be solved^16^.

Electrical stimulation (ES) is increasingly recognized as an effective therapeutic approach for promoting repair and regeneration across various damaged tissues, including nerve^17^,^18^, muscle^19^, skin^20^,^21^, and bone^22^-^24^. Specifically, low-frequency ES has been shown to accelerate axonal outgrowth and elongation, enhance the expression of neurotrophic factors, and activate key signaling pathways crucial for nerve regeneration^17^,^25^,^26^. Furthermore, ES promotes neural stem cell (NSC) proliferation^27^-^29^, migration^30^, and differentiation^27^,^31^,^32^, thereby facilitating tissue repair at injury sites^28^,^33^,^34^. ES may also relieve pain by reducing the hyperexcitability of peripheral sensory afferents, limiting the activation of primary nociceptive fibers, and attenuating inflammation-induced sensitization of dorsal horn neurons^35^-^38^. These beneficial effects have led to its widespread clinical adoption^37^. Despite these advantages, conventional ES techniques are limited by their reliance on invasive implantation procedures^39^, percutaneous wires^40^,^41^, and external power supplies^42^,^43^. These drawbacks elevate the risk of infection, tissue damage, and often necessitate secondary surgeries for electrode removal, thereby restricting the therapeutic potential and broader clinical translation of ES. Therefore, developing wireless ES systems that eliminate the need for bulky hardware is a critical objective in neural tissue engineering.

Piezoelectric biomaterials generate electrical signals in response to mechanical stress^44^-^47^, which is a key feature for their application in nerve regeneration. This property enables noninvasive ES through mechanical deformation, circumventing the need for toxic battery-based power sources. Recently, piezoelectric materials have been increasingly explored in various contexts of tissue engineering, including bone, muscle, dental tissue, skin, and neural regeneration^48^. For instance, Wu et al. fabricated a BaTiO-coated piezoelectric porous titanium alloy (Ti6Al4V) scaffold using electron beam melting (EBM) and 3D printing techniques for bone repair^49^. Similarly, another study developed an electrospun polyvinylidene fluoride (PVDF) nanofiber membrane, demonstrating that mild, deformation-induced ES can promote epidermal cell proliferation and migration to accelerate wound healing^50^. However, for neural applications requiring stringent biocompatibility, conventional piezoelectric materials such as non-degradable PVDF or potentially cytotoxic lead zirconate titanate (PZT) are often unsuitable^51^,^52^.

This has spurred growing interest in degradable piezoelectric materials that combine bioelectricity with biodegradability^53^. U.S. Food and Drug Administration (FDA)-approved poly (L-lactic acid) (PLLA) is particularly attractive due to its low cost, ease of fabrication, tunable mechanical properties, and modifiable surface functionalities^54^-^56^. PLLA is a biodegradable polymer with piezoelectric properties, and its piezoelectric response is closely associated with molecular-chain orientation, crystallinity, and crystal phase structure^57^-^59^. Mechanistically, the piezoelectricity of PLLA originates from the non-centrosymmetric arrangement of polar groups along its chiral helical chains. Carbonyl (C = O) dipoles branch from the polymer backbone, and ultrasound-induced shear strain can induce slight dipole rotation and reorientation, thereby changing chain polarization and generating piezopotential^60^. In addition, PLLA exhibits good biocompatibility and biodegradability. Compared with conventional non-degradable piezoelectric polymers or ceramic materials, PLLA is more advantageous for implantable biomedical applications^57^. PLLA-based composites have demonstrated considerable promise in bone regeneration^61^, neural repair^62^, and wound healing^63^,^64^. Nonetheless, the specific role of PLLA in managing NP following nerve injury remains largely unexplored, and its underlying therapeutic mechanisms are poorly understood.

To bridge this critical gap, we designed an injectable and fully biodegradable piezoelectric hydrogel based on PLLA. Aligned, highly crystalline PLLA nanofiber mats were fabricated via electrospinning and cryosectioned into short piezoelectric nanofibers (sPLLA), which were subsequently integrated into a methacrylated gelatin (GelMA) hydrogel matrix. The resulting composite (sPLLA-Gel) exhibits notable injectability and ultrasound responsiveness, enabling wireless ES through mechanoelectrical conversion upon ultrasonic activation. This capability eliminates the need for surgical implantation, external power sources, or secondary extraction procedures, substantially reducing infection risks and procedural trauma.

To evaluate the functional properties of the PLLA-based piezoelectric hydrogel developed in this study, we conducted a series of *in vitro* experiments, including macrophage polarization and NSC differentiation assays. The results confirmed the bioactivity of the hydrogel and revealed its underlying mechanisms of action at the cellular level. In a rat model of chronic constriction injury (CCI), behavioral tests and histopathological evaluations demonstrated that local administration of sPLLA-Gel enhanced sciatic nerve regeneration, supported remyelination and axonal growth, and markedly alleviated NP. Transcriptomic analysis further suggested that the hydrogel promotes tissue repair by modulating critical biological processes—such as cell adhesion, axon guidance, and anti-inflammatory responses—likely through regulation of transient receptor potential (TRP) channel and the NF-κB signaling pathway.

**Figure 1:**
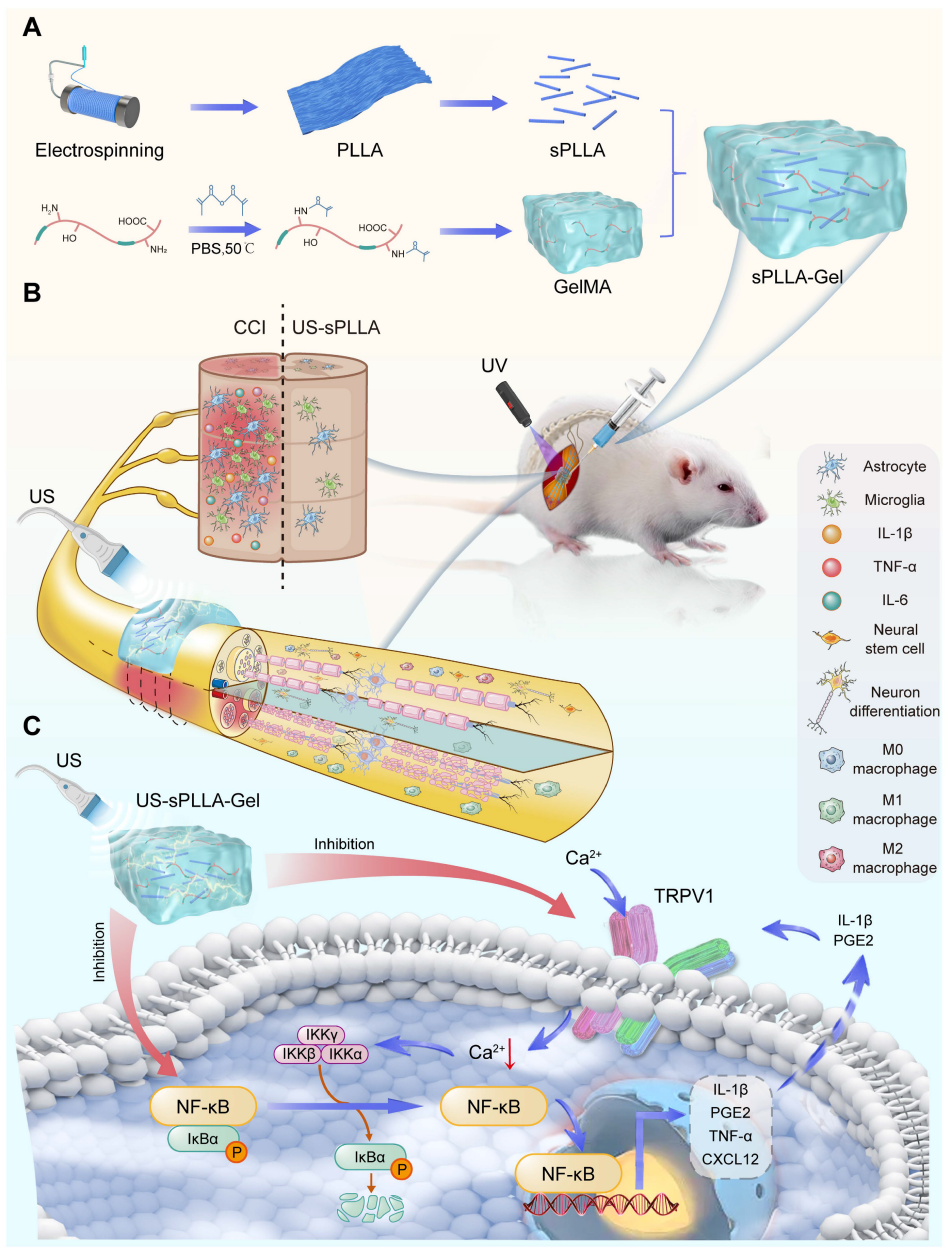
** Schematic illustration of the fabrication and therapeutic mechanism of ultrasound-responsive sPLLA-Gel hydrogel for NP caused by PNI.** (A) Fabrication of the sPLLA-Gel hydrogel. (B) Injection of the sPLLA-Gel hydrogel into the sciatic nerve injury site of a rat chronic constriction injury (CCI) model, followed by UV crosslinking and ultrasound (US) stimulation, promoted axonal and myelin regeneration, neural stem cell (NSC) differentiation toward neuron-like cells^65^,^66^, and macrophage M2 polarization, thereby facilitating nerve repair. (C) Schematic illustration of the mechanism in spinal glial cells. Ultrasound-activated sPLLA-Gel suppresses TRPV1 /NF-κB signaling and reduces pro-inflammatory mediator expression, thereby alleviating neuroinflammation and neuropathic pain (NP) caused by peripheral nerve injury (PNI).

Collectively, this work provides a comprehensive multilevel evaluation—molecular, cellular, and organismal—of the therapeutic potential of this ultrasound-activated piezoelectric hydrogel, offering not only novel mechanistic insights but also a promising treatment paradigm for the clinical management of peripheral neuropathy and associated pain conditions **(Figure 1)**.

## Results and Discussion

### Preparation and characterization of sPLLA-Gel hydrogel

**Figure 2:**
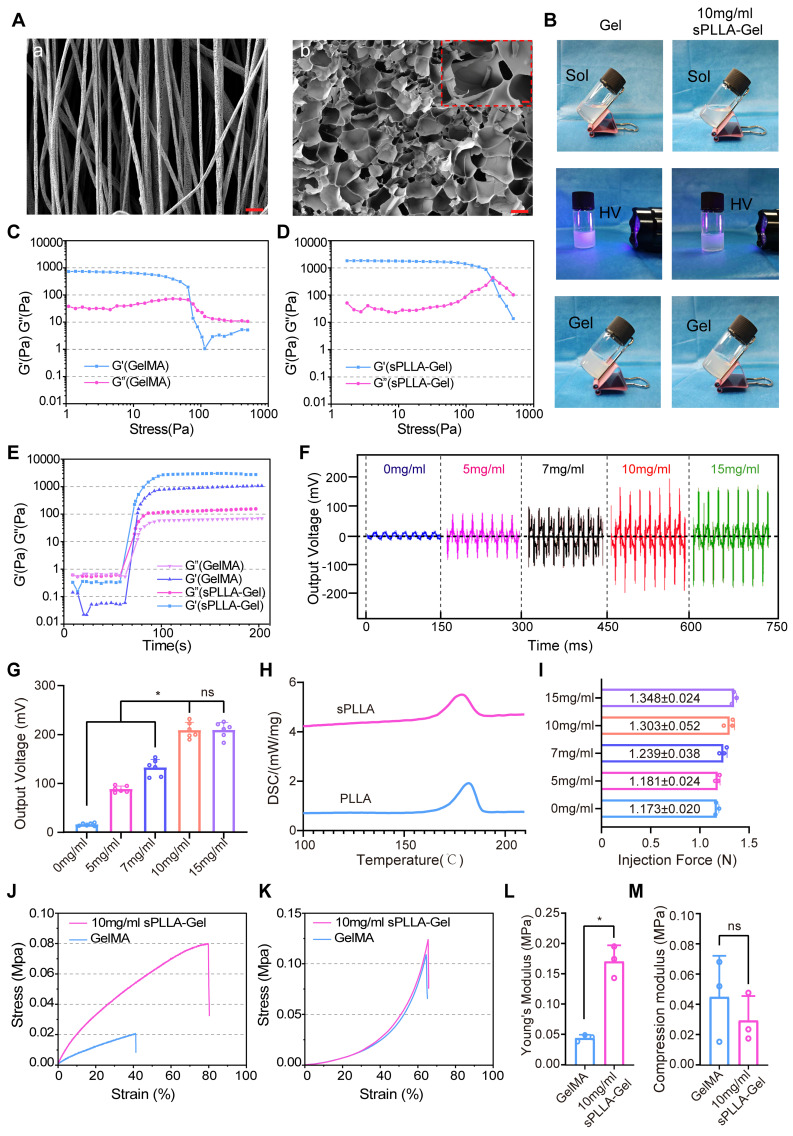
** Characterization of piezoelectric hydrogel sPLLA-Gel.** (A) SEM images. (a) Electrospun PLLA nanofiber mat (scale bar: 5 μm). (b) Cross-sectional view of freeze-dried sPLLA-Gel hydrogel (scale bar: 50 μm, inset: 5 μm). (B) Photographs showing the sol–gel transition of the hydrogel under 405 nm light irradiation. (C, D) Strain sweep tests of GelMA (C) and sPLLA-Gel (D). (E) Time sweep measurements of GelMA and sPLLA-Gel hydrogels. (F) Output voltage of oscilloscope of freeze-dried sPLLA-Gel hydrogels with varying sPLLA concentrations. (G) Statistical analysis of output voltage (n = 6). (H) DSC analysis of PLLA and sPLLA. (I) Injection force measurement of sPLLA-Gel hydrogels with different sPLLA concentrations. Stress–strain curves under tensile (n = 3). (J) and compressive (K) testing, and quantitative analysis of Young’s modulus (L) and compressive modulus (M) (n = 3). (*P < 0.05, ns means not significant (P > 0.05)).

We fabricated a uniformly aligned electrospun piezoelectric PLLA nanofiber mat using an established electrospinning technique reported previously.^67^-^69^ The mat was embedded in O.C.T. compound and cryosectioned into short fibers (designated as sPLLA), which were then incorporated into a GelMA hydrogel to form the composite sPLLA-Gel hydrogel. Scanning electron microscopy (SEM) images revealed the morphology of the electrospun PLLA mat **(Figure 2A(a))** and the distribution of sPLLA within the lyophilized hydrogel **(Figure 2A(b))**, clearly demonstrating the integration of sPLLA throughout the hydrogel.

To verify the homogeneous dispersion of sPLLA within the GelMA matrix, we labeled sPLLA with Rhodamine B and visualized the distribution via fluorescence microscopy. The results confirmed uniform dispersion of sPLLA (red fluorescence) throughout the hydrogel **(Figure S1A)**, a critical feature for establishing a continuous piezoelectric network to enhance voltage output.

Upon UV irradiation (405 nm), the hydrogel precursor rapidly crosslinked into a stable gel, indicating good photocrosslinking capability **(Figure 2B)**. To further evaluate the viscoelastic properties, structural stability, and phase transition behavior of the materials, rheological analysis was conducted. Strain sweep tests showed that sPLLA-Gel exhibited a significantly higher yield strain than GelMA, suggesting superior structural toughness and enhanced deformation resistance and mechanical stability **(Figure 2C, D)**. Time sweep analysis indicated both GelMA and sPLLA-Gel rapidly gelled within 1 minute and maintained stable storage (G′) and loss (G″) moduli, demonstrating excellent phase transition properties **(Figure 2E)**.

To determine the optimal sPLLA concentration for piezoelectric performance, sPLLA-Gel hydrogels with varying sPLLA concentrations were lyophilized to create sPLLA-Gel scaffolds. These scaffolds were placed between two aluminum foil electrodes with copper wires connected to an oscilloscope **(Figure 2F)**. Under ultrasound stimulation, both the 10 mg/mL and 15 mg/mL sPLLA-Gel hydrogels exhibited robust piezoelectric output (~200 mV), significantly exceeding the outputs of hydrogels with 0, 5, or 7 mg/mL sPLLA **(Figure 2G)**. There was no significant difference in output between the 10 and 15 mg/mL groups, indicating that once a threshold concentration is reached, sufficient fiber network formation allows effective piezoelectric response, while further increases may lead to fiber aggregation, limiting performance gains. Given that crystallinity is a key factor influencing piezoelectricity, we assessed the crystalline structure of the PLLA mat and sPLLA fibers using differential scanning calorimetry (DSC). As shown in **Figure 2H**, both materials demonstrated high crystallinity (> 70%).

We next assessed injectability, water content, and mechanical performance of the hydrogels. **Figure 2I** shows the injection force required to extrude sPLLA-Gel hydrogels with 0–15 mg/mL sPLLA concentrations through a 25G needle using a 1 mL syringe **(Figure S1A(a))**. All samples required injection forces in the range of 1.17–1.35 N, far below the upper manual injection limit of 40 N in humans, indicating excellent injectability across all tested concentrations.

To examine whether sPLLA affects water content, we measured the water content of sPLLA-Gel hydrogels with various sPLLA concentrations. Although a slight reduction was observed with increasing sPLLA concentration, all samples retained high water content (≥ 88.9 ± 1.87%), and no statistically significant differences were found compared to pure GelMA **(Figure S1B)**. These results suggest that the addition of sPLLA does not disrupt the hydrogel’s network structure or hydrophilicity. The high-water content (> 90%) also contributes to the hydrogel’s intrinsic conductivity.

Tensile and compressive tests were performed on GelMA and 10 mg/mL sPLLA-Gel hydrogels, with corresponding stress–strain curves shown in **Figure 2J and 2K**. Young’s modulus **(Figure 2L)** and compressive modulus **(Figure 2M)** were calculated. Quantitative analysis revealed a significant increase in Young’s modulus for sPLLA-Gel (~ 0.17 MPa) compared to GelMA (~ 0.045 MPa, P < 0.05), indicating improved resistance to deformation and enhanced mechanical strength. Although the compressive modulus of sPLLA-Gel was slightly lower than that of GelMA, the difference was not statistically significant, suggesting similar compressive properties between the two hydrogels.

### *In vitro* and* in vivo* degradation of sPLLA-Gel hydrogel

We evaluated the biodegradability of sPLLA-Gel hydrogels under simulated *in vitro* and *in vivo* conditions. Degradation curves indicated that all five concentrations of sPLLA-Gel hydrogels experienced approximately 40% weight loss after 28 days of incubation in PBS **(Figure S2C)**. These results suggest that the incorporation of sPLLA did not significantly alter the degradation behavior of the hydrogel, demonstrating its good stability and controllability.

Although PBS degradation studies provide valuable reference information, they cannot fully reflect the complexity of *in vivo* environments. Therefore, we further assessed *in vivo* degradation using Cy5.5-labeled GelMA and 10 mg/mL sPLLA-Gel hydrogels via fluorescence imaging in rats. After 28 days, both hydrogels exhibited substantial degradation. Quantitative analysis of the fluorescence signals showed degradation rates exceeding 50% for both groups by day 28 **(Figure S2A–B)**, which was slightly higher than the PBS results. This enhanced degradation may be attributed to the presence of various enzymes *in vivo*, such as collagenase, protease, and lysozyme, which can accelerate hydrogel breakdown. Additionally, implantation-induced mild inflammatory responses and enzyme secretion or phagocytosis by macrophages may also contribute to the degradation process. Importantly, the degradation rate of sPLLA-Gel closely matched the timeline of nerve regeneration, providing sufficient structural support for nerve cell migration, tissue regeneration, and pain relief.

### Stability of piezoelectric output of sPLLA-Gel under repeated ultrasound stimulation and *in vitro* degradation

To further evaluate the stability of the piezoelectric performance of sPLLA-Gel, we first examined the cyclic output behavior of the 10 mg/mL sPLLA-Gel samples under repeated ultrasound stimulation. As shown in **Figure S3A**, the output voltage of the 10 mg/mL sPLLA-Gel samples showed no obvious fluctuation during 1000 cycles of ultrasound activation, and no statistically significant difference was observed among the selected cycle points, indicating good cyclic piezoelectric stability. We next assessed the effect of degradation on voltage output. During 28 days of* vitro* degradation, the lyophilized 10 mg/mL sPLLA-Gel samples maintained similar output voltages at days 1, 3, 7, 14, and 28 **(Figure S3B)**. Consistently, the normalized voltage retention remained close to the initial level throughout the degradation period, with no statistically significant difference among groups **(Figure S3C)**. These results indicate that sPLLA-Gel maintains stable piezoelectric output under repeated ultrasound stimulation and during *in vitro* degradation.

### *In vitro* biocompatibility evaluation of piezoelectric hydrogel

**Figure 3:**
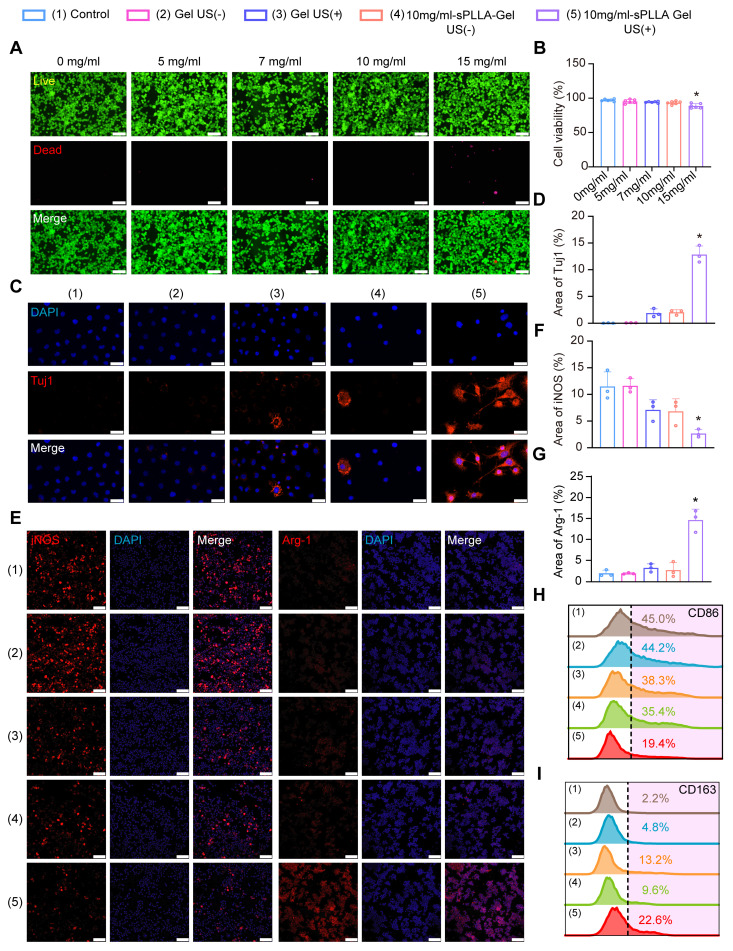
** 10 mg/mL sPLLA-Gel exhibits excellent biocompatibility, promotes NSC (C17.2) differentiation, and modulates macrophage polarization.** (A) Live/dead fluorescence staining and (B) CCK-8 assays of RSC96 cells cultured with sPLLA-Gel at various concentrations (0, 5, 7, 10 mg/mL) demonstrate good biocompatibility (n = 6). (C) Immunofluorescence staining of C17.2 cells co-cultured with hydrogels for 7 days (bar: 40 μm). (D) Quantitative analysis of the average fluorescence area of Tuj1 staining (n = 3); (E) Immunofluorescence staining of iNOS, Arg-1, and DAPI in macrophages from each group (scale bar: 100 μm). Quantitative analysis of average fluorescence areas for iNOS (F) and Arg-1 (G) (n = 3). (H, I) Flow cytometry analysis of M1 (CD86) (H) and M2 (CD163) (I) macrophage populations. (*P < 0.05, compared with all other groups).

To assess the biocompatibility of the piezoelectric hydrogel, we conducted live/dead cell staining of Schwann cells (RSC96) treated with sPLLA-Gel hydrogels at different sPLLA concentrations (0, 5, 7, 10, and 15 mg/mL) **(Figure 3A)**. Calcein-AM (green) labeled live cells, while propidium iodide (PI, red) labeled dead cells. All groups demonstrated high cell viability with minimal red fluorescence, indicating excellent cytocompatibility of the sPLLA-Gel hydrogel. At concentrations of 10 mg/mL and below, cell morphology remained intact with no signs of apoptosis or necrosis. However, in the 15 mg/mL group, a slight reduction in cell density and a mild increase in red fluorescence were observed, suggesting potential cytotoxicity at this higher concentration.

Cell viability was further quantified using the CCK-8 assay **(Figure 3B)**. Results showed no significant differences in cell viability across 0–10 mg/mL sPLLA-Gel groups (P > 0.05). In contrast, cells treated with 15 mg/mL sPLLA-Gel showed a slight but statistically significant decrease in viability. These findings were consistent with the live/dead staining results, indicating that sPLLA-Gel does not impair RSC96 cell survival or proliferation at concentrations ≤10 mg/mL but may exhibit mild cytotoxic effects at higher concentrations. Based on these results, and in combination with our oscilloscope piezoelectric output data, we selected 10 mg/mL as the optimal working concentration of sPLLA-Gel for subsequent neural differentiation experiments using C17.2 cells.

After exposure to sPLLA-Gel hydrogels of varying concentrations (0–15 mg/mL), C17.2 cells displayed a high proportion of viable cells (green) and few dead cells (red), again demonstrating good biocompatibility. However, in the 15 mg/mL group, a slight reduction in cell viability was observed, consistent with the previous CCK-8 and live/dead staining results. These findings collectively indicate that while sPLLA-Gel is safe and biocompatible at concentrations ≤ 10 mg/mL, higher concentrations may have minor cytotoxic effects. Therefore, 10 mg/mL was chosen as the optimal concentration for follow-up studies.

### sPLLA-Gel hydrogel promotes neural differentiation of C17.2 NSCs and anti-inflammatory M2 polarization of RAW 264.7 macrophages

To investigate whether sPLLA-Gel hydrogel promotes the differentiation of NSCs (C17.2), we performed immunofluorescence staining of cells co-cultured under different treatment conditions for 7 days using 4′,6-diamidino-2-phenylindole (DAPI) and β-III Tubulin (Tuj1) **(Figure 3C)**. DAPI (blue) stains cell nuclei, while Tuj1 (red) is a neuron-specific microtubule protein that marks neuronal differentiation. In the Control and Gel US (–) groups (GelMA hydrogel only), Tuj1 expression was low. The Gel US (+) group (GelMA hydrogel + ultrasound) and the 10 mg/mL sPLLA-Gel US (–) group (hydrogel without ultrasound) showed slightly elevated Tuj1 signals, but Tuj1-positive cells were few, and neuronal outgrowth was sparse and immature. These results suggest that GelMA hydrogel alone supports some adhesion of NSCs, and ultrasound may have a mild promoting effect on neural differentiation, though with limited efficiency.

In contrast, the 10 mg/mL sPLLA-Gel US (+) group (hydrogel + ultrasound) exhibited a significantly higher number of Tuj1-positive cells. Neuronal processes were more numerous, longer, and more complex, indicating a more mature neuronal phenotype. Quantitative analysis of Tuj1-positive area **(Figure 3D)** confirmed that this group had the highest percentage of neuronal marker expression. These findings suggest that 10 mg/mL sPLLA-Gel hydrogel, when activated by ultrasound, can effectively induce neural differentiation of C17.2 cells.

To further evaluate the potential anti-inflammatory effects of sPLLA-Gel hydrogel in an inflammatory microenvironment, we used an *in vitro* inflammation model in which RAW 264.7 macrophages were stimulated with LPS + IFN-γ to induce pro-inflammatory M1 polarization. The ability of the hydrogel to promote M2 polarization was assessed via immunofluorescence staining of M1 marker iNOS and M2 marker Arg-1 **(Figure 3E–G)**. In the Control and Gel US (–) groups, iNOS expression and the proportion of positive cells were high, while Arg-1 expression was low, confirming successful establishment of the inflammatory model. The Gel US (+) and 10 mg/mL sPLLA-Gel US (–) groups showed modest reductions in iNOS expression and slight increases in Arg-1 expression, although not statistically significant.

Notably, in the 10 mg/mL sPLLA-Gel US (+) group, the proportion of iNOS-positive cells was significantly reduced, while Arg-1-positive cells were significantly increased, indicating that ultrasound-activated sPLLA-Gel hydrogel promoted macrophage polarization from the M1 to the anti-inflammatory M2 phenotype.

To quantitatively validate this polarization, we performed flow cytometry (FCM) using CD86 (M1 marker) and CD163 (M2 marker) **(Figure 3H, I)**. The Control and Gel US (–) groups had the highest CD86 expression levels (45.0% and 44.2%, respectively) and lowest CD163 expression (2.2% and 4.8%). The Gel US (+) and 10 mg/mL sPLLA-Gel US (–) groups showed slightly decreased CD86 expression (38.3% and 35.4%) and increased CD163 expression (13.2% and 9.6%). However, the 10 mg/mL sPLLA-Gel US (+) group exhibited the lowest CD86 expression (19.4%) and highest CD163 expression (22.6%), both significantly different from all other groups **(Figure S4A–B)**.

We further evaluated the hydrogel’s immunomodulatory effects using RT-qPCR to measure expression of pro-inflammatory (IL-6 and IL-1β) and anti-inflammatory (TGF-β1 and IL-1Ra) cytokines. Compared with other groups, the 10 mg/mL sPLLA-Gel US (+) group showed significantly reduced IL-1β and IL-6 expression, while TGF-β1 and IL-1Ra levels were significantly upregulated **(Figure S4C–F)**. Collectively, these results demonstrate that ultrasound-activated 10 mg/mL sPLLA-Gel hydrogel markedly inhibits M1 macrophage polarization, promotes M2 polarization, and exerts potent anti-inflammatory regulatory effects.

### sPLLA-Gel hydrogel promotes motor function recovery and electrophysiological restoration after sciatic nerve injury in CCI rats

Before conducting *in vivo* functional evaluations, the biocompatibility of the sPLLA-Gel hydrogel was systematically tested. As shown in **(Figure S5)**, hematoxylin and eosin (H&E) staining of major organs (liver, heart, spleen, kidney, and lung) revealed no noticeable histopathological abnormalities among different groups, demonstrating the favorable biosafety of sPLLA-Gel for NP caused by PNI.

**Figure 4:**
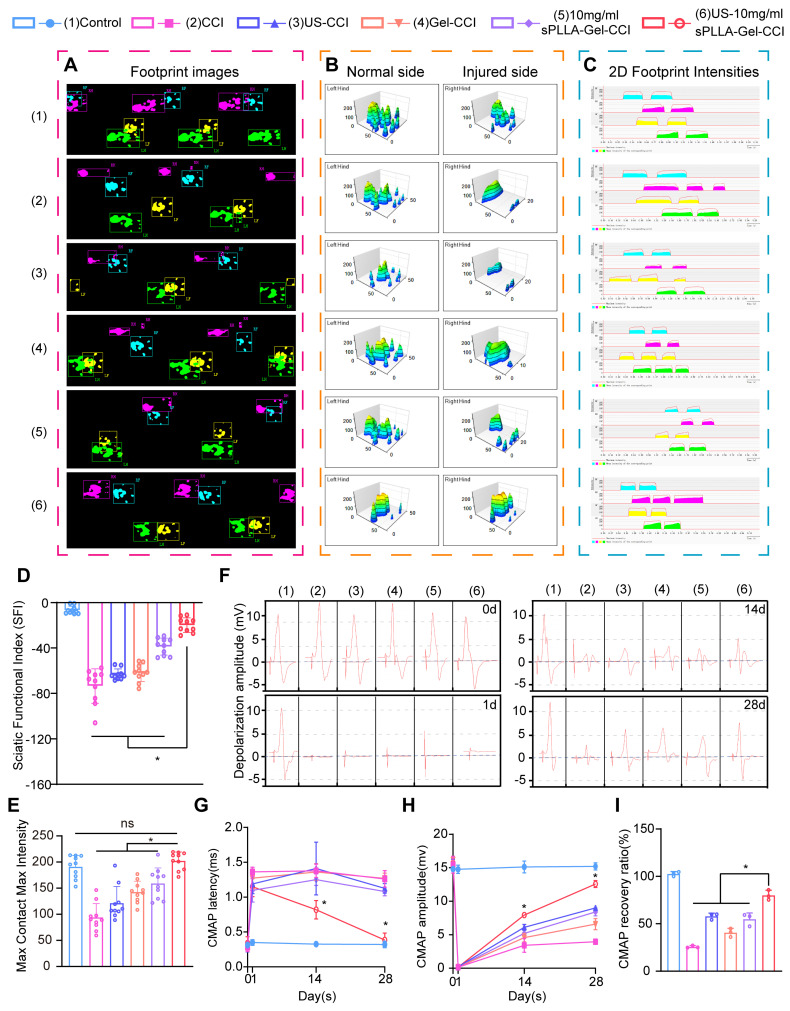
** Gait analysis and electrophysiological assessment confirmed the therapeutic effects of ultrasound-activated 10 mg/mL sPLLA-Gel on nerve repair in a rat model of sciatic nerve injury.** (A) Representative footprint images on postoperative 28 days for each group (pink: right hindlimb [RH], injured side; green: left hindlimb [LH], uninjured side). Representative 3D (B) and 2D (C) gait pressure distribution maps. (D) Statistical analysis of the sciatic functional index (SFI) at day 28 post-surgery (n = 10). (E) Maximum contact max intensity at the time of maximum contact on day 28 post-surgery (n = 10). (F) Electromyography of CMAP of the injured sciatic nerve. (G) CMAP latency (n = 3). (H) CMAP amplitude (n = 3). (I) CMAP recovery ratio (n = 3). (*P < 0.05, ns means not significant (P > 0.05)).

Gait analysis plays a key role in evaluating motor function recovery and indirectly assessing pain-related behavior after sciatic nerve injury in rats. In this study, we used multiple gait assessment methods **(Figure S6)**, including footprint imaging, two-dimensional (2D) and three-dimensional (3D) plantar pressure mapping, and quantitative analysis via the Sciatic Functional Index (SFI) and Maximum Contact Max Intensity (MCMI), to comprehensively evaluate the effects of different treatments on post-injury gait **(Figure 4A–E)**.As shown in **Figure 4A**, rats in the Control group exhibited regular gait patterns with symmetrical hind limb movements. Clear footprints were observed on both the right hind (RH, injured) and left hind (LH, uninjured) limbs, with evenly distributed plantar pressure. The 2D and 3D stress maps indicated balanced plantar pressure between both sides, reflecting intact neural and muscular function **(Figure 4B–C)**. By contrast, rats in the CCI group exhibited marked gait spasms 28 days post-surgery. The RH footprint was significantly smaller with disrupted rhythmicity, indicating impaired motor coordination due to nerve injury. The 2D and 3D pressure maps showed dramatically reduced plantar pressure on the RH side and shortened ground contact time, with force concentrated at the heel. This suggests an antalgic (pain-avoidant) gait, where the injured limb could not adequately support body weight. Rats in the US-CCI, Gel-CCI, and 10 mg/mL sPLLA-Gel-CCI groups showed mild gait improvements, with slightly increased footprint areas and modest recovery of RH morphology. However, evident dysfunction remained. The pressure distribution maps showed slightly improved force application on the injured limb but remained suboptimal. Strikingly, rats in the US-10 mg/mL sPLLA-Gel-CCI group exhibited the most substantial gait recovery among all treatment groups. Their gait pattern and RH plantar pressure were most similar to the Control group, indicating superior recovery of nerve and muscle function **(Figure 4A–C)**.

SFI, a non-invasive metric for assessing sciatic nerve recovery, showed the highest scores in the US-10 mg/mL sPLLA-Gel-CCI group, closely approaching Control levels and significantly outperforming the CCI group. While US-CCI, Gel-CCI, and 10 mg/mL sPLLA-Gel-CCI groups showed slight improvement over CCI, no statistical significance was observed (P > 0.05) **(Figure 4D)**. MCMI, which reflects the peak plantar pressure during walking, correlates with gait, neuromuscular function, and pain severity. CCI rats exhibited significantly reduced MCMI values, suggesting severe NP that caused decreased limb loading. MCMI values modestly increased in the US-CCI and Gel-CCI groups, indicating partial improvement in neural repair and pain relief, though differences remained statistically insignificant. In contrast, the US-10 mg/mL sPLLA-Gel-CCI group showed significantly higher MCMI values, approaching those of the Control group, highlighting its remarkable effect in alleviating NP **(Figure 4E)**.

To verify whether gait improvements reflected true nerve conduction and neuromuscular recovery, electrophysiological assessments of the sciatic nerve were performed at multiple time points (0, 1, 14, and 28 days post-operation). Electromyographic analysis of compound muscle action potentials (CMAP) showed significantly prolonged CMAP latency in the CCI and other untreated groups, indicating impaired conduction and demyelination^70^. In the US-10 mg/mL sPLLA-Gel-CCI group, latency was also prolonged at day 1 but progressively shortened over time, approaching Control values by day 28 **(Figure 4F–G)**. This suggests that ultrasound-activated sPLLA-Gel hydrogel significantly accelerated remyelination and restored conduction velocity.

To further assess functional reinnervation, we measured CMAP amplitude and recovery ratio. The US-10 mg/mL sPLLA-Gel-CCI group showed significantly higher CMAP amplitudes at days 14 and 28 **(Figure 4H)**, and the CMAP recovery ratio reached approximately 80% by day 28, significantly surpassing all other untreated groups **(Figure 4I)**. This indicates that by day 14, axonal regeneration and muscle reinnervation had occurred and continued to improve by day 28, approaching normal function.

Collectively, these findings demonstrate that ultrasound-activated sPLLA-Gel hydrogel effectively improves motor behavior, relieves NP, restores nerve conduction, and promotes neuromuscular function after sciatic nerve injury in rats. These results highlight its robust nerve repair potential and promising translational value.

### sPLLA-Gel hydrogel attenuates gastrocnemius muscle atrophy, fibrosis, and fatty degeneration after sciatic nerve injury and promotes nerve regeneration

**Figure 5:**
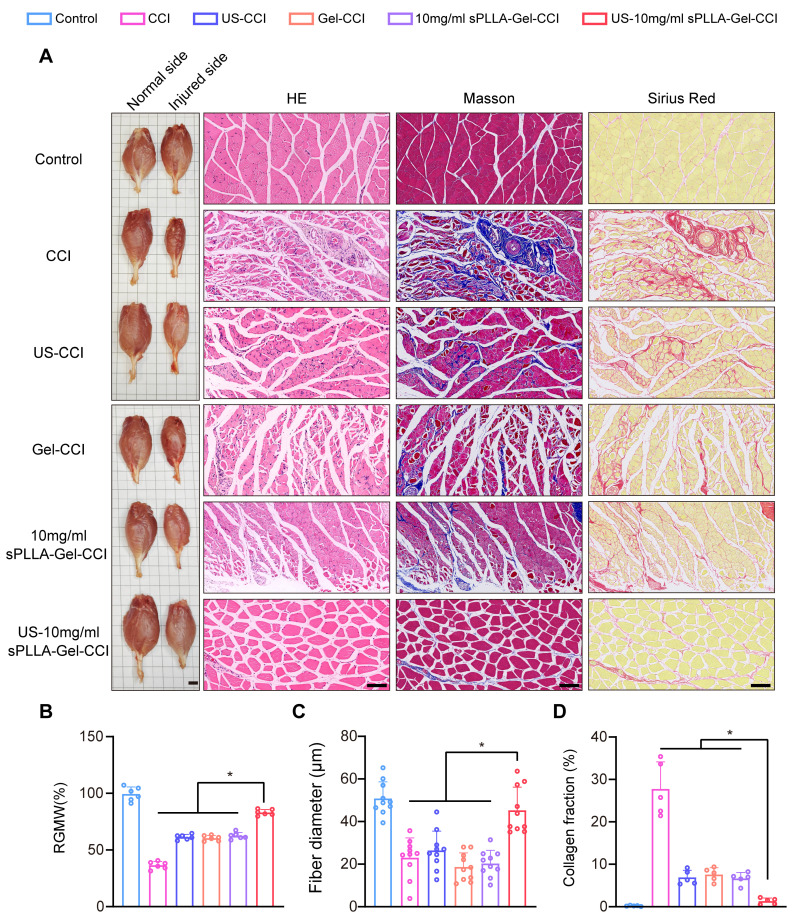
** Ultrasound-activated 10 mg/mL sPLLA-Gel alleviates gastrocnemius muscle atrophy and fibrosis induced by sciatic nerve injury at 28 days post-surgery.** (A) Representative gross images of gastrocnemius muscles (scale bar:5 mm) and H&E, Masson, and Sirius Red staining of cross-sections from the injured side (scale bar:100 μm). (B) Relative gastrocnemius muscle weight (RGMW) ratio (n = 6). (C) Quantification of gastrocnemius myofiber diameter (n = 10). (D) Proportion of collagen fiber area in gastrocnemius muscle (n = 5). (*P < 0.05.)

Muscle atrophy is a common secondary pathological change following PNI, severely affecting daily activity and quality of life^71^. The gastrocnemius muscle, one of the primary muscles innervated by the sciatic nerve, serves as a key indicator of nerve function. Following nerve injury, denervated muscles undergo varying degrees of atrophy^72^. Conversely, muscle mass can gradually recover as regenerating nerves re-establish innervation. Therefore, the degree of gastrocnemius atrophy is a critical marker for assessing sciatic nerve functional recovery. After observing significant improvements in nerve conduction with sPLLA-Gel hydrogel via electrophysiological assessment, we hypothesized that it might also alleviate muscle atrophy caused by denervation. To validate this, we dissected and collected both the ipsilateral (injured) and contralateral (uninjured) gastrocnemius muscles from each group at 4 weeks post-surgery. Gross morphology showed that all groups except the Control exhibited varying degrees of muscle atrophy. The CCI group displayed the most pronounced reduction in muscle volume, indicating severe atrophy due to nerve injury. The US-CCI, Gel-CCI, and 10 mg/mL sPLLA-Gel-CCI groups showed moderately improved muscle size compared to the CCI group, but volumes remained clearly reduced relative to the uninjured side. Notably, the US-10 mg/mL sPLLA-Gel-CCI group had the most preserved muscle volume, approaching that of the Control group **(Figure 5A)**. Relative gastrocnemius muscle wet weight (RGMW) analysis showed a significant decrease in the CCI group, while treated groups exhibited varying degrees of recovery. The US-10 mg/mL sPLLA-Gel-CCI group demonstrated a significantly higher RGMW than all untreated groups, nearing Control values, suggesting superior efficacy in mitigating muscle atrophy **(Figure 5B)**.

To further quantify muscle fiber morphology, H&E staining revealed that the CCI group had disorganized muscle fiber alignment and significantly reduced fiber diameter. Other untreated groups showed modest improvements. In contrast, the US-10 mg/mL sPLLA-Gel-CCI group exhibited significantly larger muscle fiber diameters compared to the CCI and other untreated groups **(Figure 5C)**, indicating that ultrasound-activated sPLLA-Gel hydrogel help prevent muscle fiber degeneration. Masson and Sirius Red staining **(Figure 5A)** showed markedly increased collagen deposition in the CCI group, whereas the US-10 mg/mL sPLLA-Gel-CCI group had significantly reduced fibrosis compared to untreated groups, suggesting effective inhibition of post-injury fibrotic remodeling. To assess fatty infiltration in the gastrocnemius muscle, Oil Red O staining was performed **(Figure S7A)**. Control group muscles showed minimal lipid deposition, while the CCI group exhibited numerous red lipid droplets, indicative of fatty degeneration due to muscle denervation. Untreated groups also showed prominent lipid accumulation. However, the US-10 mg/mL sPLLA-Gel-CCI group exhibited significantly fewer lipid droplets and reduced lipid area **(Figure S7B)**, with more intact muscle architecture, suggesting this treatment may effectively attenuate denervation-induced fatty infiltration.

**Figure 6:**
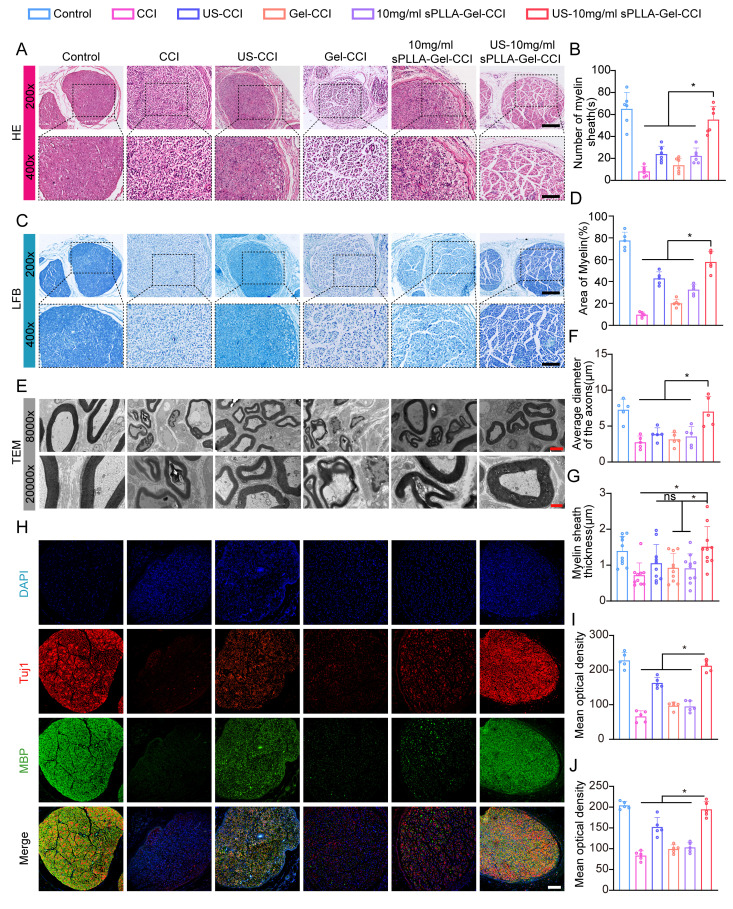
** Histopathological and ultrastructural evaluation confirms the therapeutic efficacy of the hydrogel in promoting sciatic nerve regeneration at 28 days post-operation.** (A) H&E staining of sciatic nerve cross-sections in each group (scale bars: 200 μm at 200×; 100 μm at 400×). (B) Number of myelinated fiber (from H&E) (n = 6). (C) LFB staining of sciatic nerve cross-sections to assess myelin integrity (scale bar: 200×, 200 μm; 400×, 100 μm). (D) Mean stained area of myelin sheaths (from LFB staining) (n = 5). (E) Representative transmission electron microscopy (TEM) images illustrating ultrastructural details (scale bars: 3 μm at 8,000×; 1 μm at 20,000×). (F) Mean diameter of myelinated axons (from TEM) (n = 5). (G) Myelin sheath thickness assessed (from TEM) (n = 10). (H) Immunofluorescence staining for myelin basic protein (MBP, red) and βIII-tubulin (Tuj1, green) (scale bar: 100 μm). (I) Tuj1 fluorescence intensity (n = 5). (J) MBP fluorescence intensity (n = 5). (*P < 0.05, ns means not significant (P > 0.05)).

To evaluate nerve regeneration, histological analysis of sciatic nerve cross-sections was performed at postoperative day 28. H&E staining revealed disorganized nerve fibers, disrupted myelin, and reduced axon density in the CCI group compared to Control. Compared with the CCI group, the US-CCI group showed partial improvement in nerve fiber organization and myelin morphology, whereas the Gel-CCI group and 10 mg/mL sPLLA-Gel-CCI group exhibited only mild histological improvement. The US-10 mg/mL sPLLA-Gel-CCI group exhibited more organized neural structure and significantly increased myelin density **(Figure 6A, B)**. Luxol Fast Blue (LFB) staining further supported these findings. The US-CCI group showed an increased myelin-stained area compared with the CCI group, whereas the Gel-CCI group and 10 mg/mL sPLLA-Gel-CCI group displayed only modest recovery trends. Notably, the US-10 mg/mL sPLLA-Gel-CCI group had the largest myelin-stained area, outperforming all untreated groups **(Figure 6C, D)**, indicating enhanced myelin regeneration. Transmission electron microscopy (TEM) **(Figure 6E)** showed severely damaged and thinned myelin sheaths in the CCI group. The US-CCI group showed partial improvement in myelin morphology, whereas the Gel-CCI group and 10 mg/mL sPLLA-Gel-CCI group demonstrated only limited restoration of myelin structure. In contrast, the US-10 mg/mL sPLLA-Gel-CCI group preserved intact myelin structures, with significantly increased axon diameter **(Figure 6F)** and myelin thickness **(Figure 6G)** compared to untreated groups. Immunofluorescence staining for Tuj1 (a neuronal marker) and MBP (myelin basic protein) revealed weak signals in the CCI group. Among the treatment groups, the US-CCI group showed a moderate increase in Tuj1 and MBP staining, while the Gel-CCI group and 10 mg/mL sPLLA-Gel-CCI group showed only slight increases compared with the CCI group. The US-10 mg/mL sPLLA-Gel-CCI group exhibited the strongest fluorescence intensities of both Tuj1 and MBP **(Figure 6H–J)**, indicating improved axonal regeneration and remyelination.

In summary, ultrasound-activated sPLLA-Gel hydrogel significantly promotes myelin repair, improves axonal morphology, and enhances nerve regeneration. These findings highlight its strong therapeutic potential for PNI repair.

### sPLLA-Gel hydrogel alleviates NP by suppressing spinal glial cell activation and inflammatory cytokine expression

**Figure 7:**
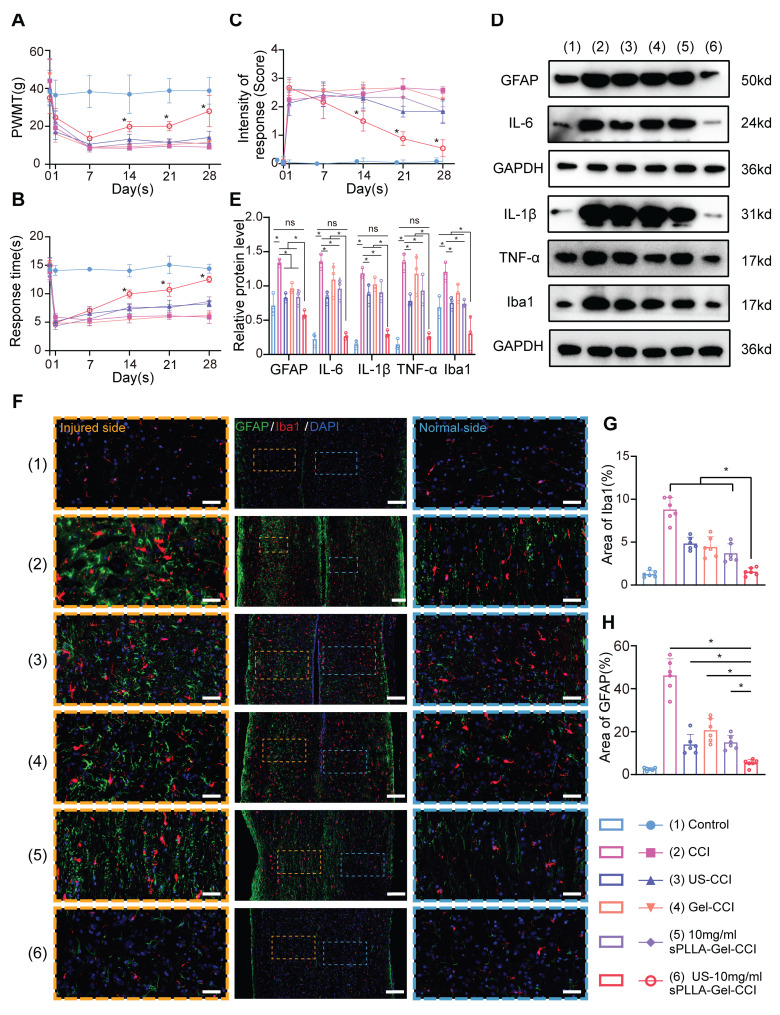
** Behavioral and molecular evidence demonstrates that Ultrasound-activated 10 mg/mL sPLLA-Gel alleviates NP in rats at 28 days post-surgery.** (A) Paw withdrawal mechanical threshold (PWMT) test (n = 6). (B) Hot plate test (n = 6). (C) Acetone test (n = 6). (D) Western blot analysis of key proteins associated with NP signaling. (E) Densitometric quantification of protein expression levels (n = 3). (F) IF staining of spinal cord for GFAP (astrocyte marker) and Iba1 (microglial marker) (central panel scale bar: 200 μm, flanking scale bars:50 μm). (G) Semi-quantification analysis of Iba1 fluorescence intensity (n = 6). (H) Semi-quantification of GFAP fluorescence intensity (n = 6). (*P < 0.05).

To comprehensively evaluate the therapeutic potential of sPLLA-Gel in NP, we conducted multi-dimensional assessments **(Figure S5)**. As shown in the behavioral tests **(Figure 7A)**, the paw withdrawal mechanical threshold (PWMT) values of all experimental groups significantly decreased within the first 7 days post-surgery compared to the Control group, indicating pronounced mechanical allodynia induced by CCI. From day 14 to day 28, PWMT values in the US-10 mg/mL sPLLA-Gel-CCI group progressively increased, reaching the highest level on the 28th day. In contrast, PWMT values in the untreated groups (e.g., US-CCI, Gel-CCI, and sPLLA-Gel-CCI) showed only slight increases and did not significantly differ from the CCI group, suggesting that ultrasound or unloaded hydrogel alone has limited impact on mechanical allodynia. In the hot plate test **(Figure 7B)**, the response latency to thermal stimuli was markedly reduced in all groups compared to the Control group. From day 7 post-surgery, the CCI group exhibited persistently shortened response latencies through day 28, reflecting sustained thermal hyperalgesia. In contrast, the US-10 mg/mL sPLLA-Gel-CCI group showed a gradual extension in thermal response latency from day 14 to 28, with significantly improved thresholds compared to other untreated groups. Similar trends were observed in the acetone test for cold allodynia **(Figure 7C)**. The cold response scores in the US-10 mg/mL sPLLA-Gel-CCI group significantly decreased over time starting from day 1, and by day 28, the scores were significantly lower than those of other untreated groups and approximated the Control group levels, indicating maximal relief of cold hypersensitivity. Collectively, these results demonstrate that sPLLA-Gel hydrogel significantly alleviates mechanical, thermal, and cold hypersensitivity associated with NP.

Previous studies have shown that astrocyte (GFAP) and microglia (Iba1) activation in the spinal cord plays a central role in the maintenance of NP following PNI^73^-^75^. These activated glial cells secrete pro-inflammatory cytokines such as IL-1β, TNF-α, IL-6, and IFN-γ, which are critical contributors to central sensitization^76^,^77^. To explore the underlying mechanism of pain relief by sPLLA-Gel, we systematically assessed the expression of GFAP, Iba1, and inflammatory cytokines in the spinal cord. Western blot (WB) analysis confirmed glial activation and neuroinflammation after CCI. While interventions such as US-CCI, Gel-CCI, and sPLLA-Gel-CCI slightly reduced glial activation and cytokine expression, levels remained significantly elevated compared to Control. Notably, in the US-10 mg/mL sPLLA-Gel-CCI group, the expression of GFAP and Iba1 was markedly reduced, and levels of IL-6, IL-1β, and TNF-α were significantly downregulated, approaching those observed in the Control group **(Figure 7D–E)**. Immunofluorescence staining further illustrated these findings** (Figure 7F)**. In the spinal dorsal horn (left panels with orange borders for injured sides and right panels with blue borders for normal sides), the CCI group displayed markedly increased GFAP (green) and Iba1 (red) signals on the injured side compared to the uninjured side. In contrast, the US-10 mg/mL sPLLA-Gel-CCI group exhibited significantly reduced GFAP and Iba1-positive areas on the injured side compared to untreated groups **(Figure 7H)**, consistent with the WB results.

Taken together, these findings indicate that ultrasound-activated sPLLA-Gel hydrogel effectively suppresses spinal glial cell activation and the release of pro-inflammatory cytokines, thereby mitigating central sensitization and exerting therapeutic effects at the molecular level against NP.

### Ultrasound-activated sPLLA-Gel hydrogel alleviates NP by inhibiting TRPV1 channel activation and the NF-κB signaling pathway

**Figure 8:**
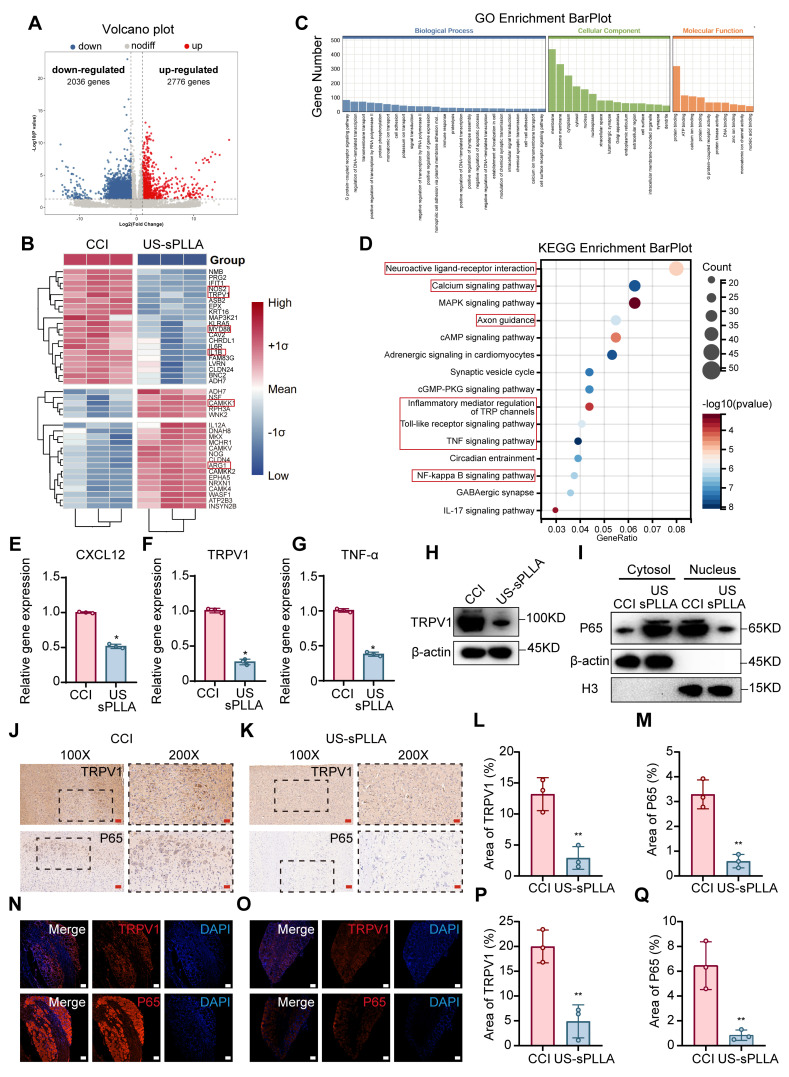
**Transcriptome sequencing reveals the biological mechanism of ultrasound-activated sPLLA-Gel in modulating NP.** (A) Volcano plot of differentially expressed genes (DEGs) between CCI and US-sPLLA groups. (B) Heatmap of DEGs between the two groups. (C) Gene Ontology (GO) enrichment analysis of biological processes associated with significant DEGs. (D) Representative KEGG pathways enriched among DEGs. (E-G) qRT-PCR analysis of TNF-α, CXCL12, and TRPV1 mRNA levels (n = 3). (H) Western blot analysis of spinal cord tissue from CCI and US-sPLLA groups. (I) WB of nuclear and cytoplasmic protein fractions in spinal cord tissue. (J-K) Immunohistochemical staining of TRPV1 and P65 in the spinal cord tissue from CCI and US-sPLLA groups (scale bars:100 μm at 100×; 50 μm at 200×). (L-M) Semi-quantification of the positive area for TRPV1 and p65 (n = 3). (N-O) IF images of DRG stained for TRPV1 and p65 (red), with DAPI (blue) (scale bar: 100 μm). (P-Q) Semi-quantification of mean fluorescence intensity for TRPV1 and p65 (n = 3). (*P < 0.05, **P < 0.01).

To further explore the underlying mechanism by which ultrasound-activated sPLLA-Gel hydrogel alleviates NP, we performed high-throughput RNA sequencing (RNA-seq) on spinal cord tissues from the CCI group and the US-10 mg/mL sPLLA-Gel-CCI group (hereafter referred to as the US-sPLLA group). Volcano plot analysis revealed that, compared to the CCI group, 2,776 genes were significantly upregulated and 2,036 genes were significantly downregulated in the US-sPLLA group **(Figure 8A)**. The heatmap demonstrated distinct gene expression profiles between groups, particularly for inflammation- and pain-related genes such as IL-1β, MYD88, TRPV1, NOS2, and ARG1 **(Figure 8B)**. Gene Ontology (GO) enrichment analysis indicated that differentially expressed genes (DEGs) were significantly involved in several biological processes including transmembrane transport, cell adhesion, immune responses, signal transduction, and protein phosphorylation. At the Cellular Component level, DEGs were predominantly enriched in plasma membrane, cytoplasm, nucleus, neuronal synapses, and dendrites, suggesting their involvement in synaptic transmission, intracellular signaling, and nuclear transcriptional regulation. At the Molecular Function level, key enriched terms included protein binding, ATP binding, calcium ion binding, and G protein-coupled receptor activity.

These enrichments collectively support the hydrogel’s multifaceted regulatory roles in pain transmission, neuroinflammation, and neuronal function **(Figure 8C)**. Kyoto Encyclopedia of Genes and Genomes (KEGG) pathway enrichment analysis further revealed that DEGs were enriched in several canonical neurological and inflammatory pathways, including Neuroactive ligand–receptor interaction, Calcium signaling, Axon guidance, Inflammatory mediator regulation of TRP channels, Toll-like receptor signaling, NF-κB signaling, as well as TNF and IL-17 pathways **(Figure 8D)**. Notably, the enrichment of Inflammatory mediator regulation of TRP channels and NF-κB signaling pathways was consistent with the heatmap expression trends of TRPV1, IL-1β, MYD88, and ARG1, suggesting that these pathways play a central role in CCI-induced neuroinflammation, and may be suppressed by ultrasound-activated sPLLA-Gel to exert anti-inflammatory and analgesic effects.

To validate these findings, we modeled inflammatory activation in BV2 microglial cells using IL-1β and PGE2 stimulation. qRT-PCR results showed significant upregulation of TNF-α **(Figure S8A)**, CXCL12 **(Figure S8B)**, and TRPV1 **(Figure S8C)** mRNA levels, with the combined treatment showing the most pronounced effect. WB analysis further confirmed these results **(Figure S8D)**, indicating that TRPV1 is a critical ion channel mediating pain sensitization under inflammatory conditions^78^,^79^.

To determine whether the NF-κB pathway was involved, we conducted nuclear-cytoplasmic fractionation and Western blot analysis in BV2 cells. Results revealed that IL-1β and PGE2 stimulation promoted nuclear translocation of the p65 subunit, with maximal nuclear accumulation observed under co-treatment** (Figure S8E)**, confirming co-activation of the NF-κB pathway.

*In vivo* experiments demonstrated that US-sPLLA treatment significantly reduced spinal cord levels of PGE2 **(Figure S8F)** and IL-1β **(Figure S8G)** compared to CCI controls. Additionally, the mRNA expression levels of CXCL12 **(Figure 8E)**, TRPV1 **(Figure 8F)**, and TNF-α **(Figure 8G)** were markedly decreased, and TRPV1 protein expression was also significantly lower in the US-sPLLA group **(Figure 8H)**. Furthermore, nuclear-cytoplasmic fractionation of WB experiments further revealed that p65 was predominantly localized in the nucleus in the CCI group, whereas in the US-sPLLA group, p65 remained primarily in the cytoplasm **(Figure 8I)**.

To further investigate the spatial expression of TRPV1 and the NF-κB core molecule p65, immunohistochemical staining was performed on spinal cord and dorsal root ganglion (DRG) tissues. In the CCI group, both TRPV1 and p65 exhibited strong positive staining in the dorsal horn of the spinal cord and DRG **(Figure 8J, N)**. In contrast, their expression was markedly reduced in the US-sPLLA group **(Figure 8K, O)**. Semi-quantitative analysis confirmed a significant decrease in the positive staining areas of TRPV1 and p65 in the US-sPLLA group compared to the CCI group **(Figure 8L, M, P, Q)**. These results further demonstrate that the US-sPLLA hydrogel suppresses TRPV1 expression and NF-κB pathway activation at both spinal and DRG levels, leading to a reduction in downstream inflammatory mediators and the alleviation of NP. This is consistent with the *in vitro* findings. In conclusion, the inflammatory microenvironment induced by IL-1β and PGE2 activates TRPV1 and the NF-κB pathway, contributing to NP. Ultrasound-activated sPLLA-Gel hydrogel effectively inhibits this signaling cascade, thereby attenuating inflammation-induced NP following nerve injury.

## Methods

### Preparation of injectable piezoelectric PLLA nanofiber hydrogel

Methacrylated gelatin (GelMA) and photoinitiators (Phenyl-(2,4,6-trimethylbenzoyl)-lithium phosphate, LAP) were purchased from Engineering For Life (Suzhou, China). PLLA was obtained from Aladdin (Shanghai, China). N, N-Dimethylformamide (DMF, 227056) and dichloromethane (DCM, 270997) were sourced from Millipore Sigma (Burlington, MA, USA). O.C.T. was purchased from Sakura Finetek (California, USA). A cryostat microtome was provided by RWD (Shenzhen, China), and 0.22 μm microporous membranes were obtained from BKMAM (Changde, China). PLLA nanofibers were fabricated via electrospinning, following previously reported protocols^68^. Briefly, 0.8 g of PLLA was dissolved in a mixed solvent of DMF and DCM (v/v = 1:4). The solution was loaded into a syringe and electrospun using a G22 needle under an applied voltage of 14 kV and a flow rate of 2 mL/h. The electrospun fibers were collected on a rotating aluminum drum at 3300 rpm to obtain uniaxially aligned nanofiber mats. The electrospinning was conducted under controlled humidity (30–40%). The collected nanofiber mats were then annealed at 105 °C for 10 h in an oven and subsequently cooled to room temperature. A second annealing step was performed at 160.1 °C for another 10 h to further enhance crystallinity. After cooling, the nanofiber mats were embedded in O.C.T. compound and frozen at -80 °C for 2 h. The frozen blocks were then cryosectioned perpendicular to the fiber orientation using a cryostat to obtain 25 μm-long microsegments. These electrospun sPLLA were collected in centrifuge tubes containing deionized water and washed five times using 0.22 μm filters. After air drying at room temperature, sPLLA powders were obtained. The dried sPLLA was weighed and dispersed into PBS to achieve final concentrations of 0, 5, 7, 10, and 15 mg/mL. LAP was added at a concentration of 0.25% (w/v), followed by the addition of GelMA lyophilized powder at a concentration of 20% (w/v). The mixture was vortexed at 60 °C in the dark for 30 minutes until GelMA was fully dissolved. The resulting prepolymer solution was then photocrosslinked under 405 nm light irradiation for 2 minutes to form the final injectable sPLLA-Gel hydrogels.

### Materials characterization

#### SEM analysis

The sPLLA-Gel hydrogel was lyophilized using a freeze-dryer to obtain the dehydrated scaffold. Both the lyophilized hydrogel and the pristine PLLA electrospun membrane were mounted onto conductive adhesive tape, followed by sputter-coating with gold for 45 sec at 10 mA using a Quorum SC7620 sputter coater. Subsequently, the surface morphology of the samples was observed using a field-emission scanning electron microscope (FE-SEM, ZEISS Sigma 300, Germany).

#### Measurement of open-circuit output voltage under ultrasound activation

sPLLA-Gel samples of varying concentrations were lyophilized and cut into squares measuring 1 cm×1 cm. Both sides of each sample were covered with aluminum foil of equal dimensions and adhered using conductive adhesive. Two copper wires of identical length were affixed to the aluminum foils on each side using polyimide tape. The wires were then connected to an oscilloscope (UNI-T, China), and ultrasound stimulation was applied using a therapeutic ultrasound device (BH200, Survow, China). The open-circuit voltage output was recorded in real-time. Previous studies have demonstrated that ultrasound intensities exceeding 0.5 W/cm² can induce significant thermal effects^80^. To prevent potential tissue damage while ensuring sufficient pressure threshold for piezoelectric hydrogel activation, we selected an ultrasound parameter of 1 MHz at 0.5 W/cm² for all experiments. In addition, 1 MHz ultrasound is commonly used for relatively deeper soft tissues and was therefore considered suitable for stimulation of the hydrogel implanted around the sciatic nerve *in vivo*. The same ultrasound parameters were used for both *in vitro* voltage measurement and *in vivo* treatment^81^,^82^.

#### Mechanical property characterization

Compressive Modulus Testing: Hydrogel samples (n = 3 per group) were prepared in cylindrical shapes with a diameter of 10 mm and a height of 4 mm. Each sample was mounted onto the fixture and tested using a universal testing machine (CMT6103, MTS Industrial Systems, China). The initial contact force was set to 0.01 N, and the compression rate was maintained at 5 mm/min. During testing, the force–displacement data were recorded, and the compressive modulus was calculated based on the resulting slope in the linear elastic region of the stress–strain curve.

Young’s Modulus Testing: To assess the tensile properties, samples (n = 3 per group) were fabricated into cylindrical rods with a diameter of 6 mm and a length of 5 cm. Each sample was clamped and tested using the same universal testing machine (CMT6103, MTS Industrial Systems, China). The initial force was set to 0.01 N, and the tensile speed was fixed at 5 mm/min. The force–displacement data were recorded throughout the tensile process to generate stress–strain curves, from which Young’s modulus was calculated using the slope of the linear region.

#### Rheological characterization

The rheological properties of the hydrogels were evaluated using a rotational rheometer (HAAKE MARS 40, Thermo Fisher company, USA) equipped with a parallel plate geometry (20 mm diameter, 1 mm gap), with the sample temperature maintained at 37 °C. The storage modulus (G′) and loss modulus (G″) of the cured hydrogels were measured using strain sweep tests performed at 1 Hz over a strain range from 0.1% to 1000%. The adjustment and sampling time were both set to 3 s, with 10 points per decade. This protocol represents a standard approach for assessing the rheological behavior of hydrogels in clinical applications. To monitor the curing kinetics, 200 μL of uncured GelMA precursor solution was subjected to time-dependent rheological analysis during UV light-initiated crosslinking. The evolution of storage modulus (G') during polymerization was recorded using the aforementioned parameters, providing quantitative assessment of the hydrogel's gelation profile.

#### DSC

Uniform samples (10 mg) were carefully trimmed to appropriate dimensions and placed in aluminum oxide DSC crucibles. The samples were gently flattened to ensure optimal thermal contact with the crucible base. Each crucible was hermetically sealed using a crimping press to prevent sample volatilization or oxidation during testing. Thermal analysis was conducted using a calibrated differential scanning calorimeter (DSC2500, TA DISCOVERY, USA). The heating rate was set to 10 °C/min, with the temperature ramped from 30 °C to 200 °C under a nitrogen atmosphere at a flow rate of 50 mL/min, which served to prevent sample oxidation or thermal degradation.

#### Injectability test of hydrogel

Hydrogel samples were loaded into 1 mL syringes, and air bubbles were carefully removed to avoid interference with subsequent pressure measurements. A 25G fine-gauge injection needle was attached to the syringe, which was then securely mounted on the clamp of a Mini-Biomechanical Testing Device (HRJ Inc., Jinan). Care was taken to ensure that the syringe remained stable without displacement or rotation during vertical compression. During the test, the plunger was depressed at a constant rate of 1 cm/min to simulate the typical speed of manual injection, and compression was continued until the hydrogel was fully extruded. The maximum extrusion force required during the process was recorded by the device. Each group of materials was tested in triplicate, and the mean value and standard deviation were calculated.

#### Water content of the hydrogel

The prepared sPLLA-Gel hydrogels with varying concentrations were photopolymerized in molds under 405 nm UV light. After curing, surface moisture was removed using filter paper, and the wet weight (W_0_) was measured. Samples were then freeze-dried in a vacuum freeze-dryer at -80 °C for 24 h, and the dry weight (W_1_) was recorded. The water content was calculated using the formula: Water content (%) =[(W_0_-W_1_)/W_0_] ×100%. Each group was tested in triplicate.

#### Cyclic stability of piezoelectric output under repeated US stimulation

Lyophilized 10 mg/mL sPLLA-Gel samples were prepared using the same procedure described for the initial open-circuit voltage measurement and cut into squares measuring 1 cm × 1 cm. Both sides of each sample were covered with aluminum foil of equal dimensions and connected to an oscilloscope using copper wires. Ultrasound stimulation was applied using the same parameters as those used in the *in vivo* experiments (1 MHz, 0.5 W/cm²). The output voltage was continuously recorded during 1000 cycles of repeated ultrasound stimulation. The peak open-circuit voltage was extracted at the 1st, 100th, 300th, 500th, 700th, and 1000th cycles. All experiments were performed in triplicate.

#### *In vitro* degradability and output voltage measurement of the hydrogel

Lyophilized hydrogel samples were weighed (initial dry weight, W₀) and immersed in 2 mL PBS buffer (pH 7.4) containing 1 mg/mL lysozyme at 37 °C. The PBS solution was refreshed every 48 hours to maintain consistent enzymatic activity. At predetermined intervals (days 1, 3, 7, 14, and 28), samples were retrieved, rinsed with deionized water, lyophilized, and reweighed (W₁). The remaining mass percentage was calculated as: Weight remaining (%) = W1/W0×100%. At the same time points, the lyophilized samples were further subjected to output voltage measurement under the same ultrasound conditions used for the initial piezoelectric characterization, and the voltage output at each time point was compared with that of day 0 to evaluate whether hydrogel degradation affected its piezoelectric performance. All experiments were performed in triplicate.

#### *In vitro* degradability of the hydrogel

The *in vivo* biodegradation behavior of the hydrogel was monitored using fluorescence labeling combined with live animal imaging. GelMA solution was conjugated with Cy5.5-NHS ester for fluorescent labeling. Adult male Sprague-Dawley rats (250-300 g) were anesthetized, and the sciatic nerve was exposed under sterile conditions. A total of 100 μL of Cy5.5-labeled 10 mg/mL sPLLA-Gel hydrogel was injected around the sciatic nerve, followed by immediate photo-crosslinking using 405 nm UV light and surgical wound closure. Fluorescence imaging was conducted on postoperative days 1, 3, 7, 14, and 28 using an *in vivo* imaging system (AniView Kirin, China) with an excitation wavelength of 675 nm and an emission wavelength of 740 nm. Imaging parameters, including exposure time, field of view, and fluorescence threshold, were kept consistent across all time points. Fluorescence intensity was quantified using AniView Pro software, with a minimum of three animals per group.

### *In vitro* experiments

#### Cell culture

RSC96 cells (ZQ0154, Shanghai Zhong Qiao Xin Zhou Biotechnology Co., Ltd.) and C17.2 cells (SNL-536, Wuhan Shine Biotechnology Co., Ltd.) were cultured in standard Dulbecco’s Modified Eagle Medium (DMEM, Gibco, Australia) supplemented with 10% fetal bovine serum (SP01002-0050, Sperikon Life Science & Biotechnology Co., Ltd.) and 1% penicillin–streptomycin (P/S, SP00303-0100, Sperikon Life Science & Biotechnology Co., Ltd.). RAW264.7 cells were maintained in RPMI-1640 medium (Gibco, Australia) containing 10% FBS and 1% P/S under standard conditions.

#### *In vitro* biocompatibility test

sPLLA-Gel hydrogels (0, 5, 7, 10, and 15 mg/mL) were aseptically immersed in complete culture medium (high-glucose DMEM supplemented with 10% FBS and 1% P/S) and incubated at 37 °C for 24 h. The resulting extracts were filtered through a 0.22 μm sterile membrane for further use. RSC96 cells were seeded uniformly into 96-well plates and cultured for 24 hours. The culture medium was then replaced with hydrogel extracts of varying concentrations (0, 5, 7, 10, and 15 mg/mL), followed by incubation for another 24 hours. Cell viability was assessed using a CCK-8 assay kit (C0037, Beyotime, China). After removing the medium, 10 μL of CCK-8 solution was added to each well, and the plates were incubated for 2 hours before measuring the absorbance at 450 nm using a microplate reader. For cytotoxicity evaluation, a Calcein-AM/PI double staining assay (C1371S, Beyotime, China) was employed. RSC96 cells were seeded in 6-well plates and incubated for 24 hours, followed by replacement of the culture medium with hydrogel extracts at concentrations of 0, 5, 7, 10, and 15 mg/mL. After 48 hours of incubation, cells were stained with Calcein-AM and propidium iodide (PI) for 20 minutes. Fluorescent images were acquired using a fluorescence microscope to evaluate live (green) and dead (red) cell populations.

#### NSC differentiation

The mouse neural progenitor cell line C17.2 was used to evaluate the neurogenic differentiation potential of the 10 mg/mL sPLLA-Gel hydrogel under ultrasound stimulation. Under sterile conditions, 300 μL of 10 mg/mL sPLLA-Gel solution was added to each well of a 24-well plate, ensuring uniform coverage of the well bottom. The hydrogel was then photo-crosslinked using ultraviolet light (405 nm). C17.2 cells were seeded on the surface of the hydrogel at a density of 1 × 10³ cells per well. Ultrasound activation (1 MHz, 0.5 W/cm²) was applied once daily for 10 minutes over a 7-day period. After stimulation, cells were fixed and subjected to immunofluorescence staining to detect neuronal differentiation using the marker β-Tubulin III (1:1000, ab18207, Abcam). Cell nuclei were counterstained with DAPI. Quantification of fluorescence intensity was performed using ImageJ software (NIH, Bethesda, MD, USA) to assess the extent of neuronal differentiation.

#### *In vitro* macrophage polarization and expression of inflammation-related genes

RAW264.7 cells were employed to investigate the effect of 10 mg/mL sPLLA-Gel hydrogel, under ultrasound stimulation, on macrophage polarization. A Transwell co-culture system was established by first seeding RAW264.7 cells—pre-polarized to the M1 phenotype by treatment with lipopolysaccharide (LPS, 100 ng/mL) and interferon-γ (IFN-γ, 20 ng/mL) for 24 h—in the lower chamber. GelMA hydrogel or 10 mg/mL sPLLA-Gel hydrogel was placed in the upper chamber. During co-culture, the experimental group was subjected to ultrasound stimulation (1 MHz, 0.5 W/cm², 10 min/day) for 3 consecutive days. Following co-culture, lower chamber cells were collected for analysis. Flow cytometry was used to assess macrophage polarization by staining surface markers CD86 (M1 phenotype) and CD163 (M2 phenotype) with anti-CD86 APC and anti-CD163 APC antibodies (Invitrogen, USA). Additionally, the mRNA expression levels of pro-inflammatory genes (IL-6 and IL-1β) and anti-inflammatory genes (TGF-β1 and IL1Ra) were quantified using RT-qPCR. Primer sequences are listed in **Table S1**. For immunofluorescence analysis, RAW264.7 cells were fixed with 4% paraformaldehyde (PFA) for 10 min and stained with antibodies against iNOS (1:200, ab178945, Abcam) and Arg-1 (1:200, 93668, CST), followed by nuclear counterstaining with DAPI. Images were acquired using a confocal laser scanning microscope (IXplore IX85 Spin, Olympus, Japan), and fluorescence intensity was semi-quantitatively analyzed using ImageJ software.

### Rat sciatic nerve injury pain model

Seven-week-old male Sprague-Dawley rats (200-300 g), specific pathogen-free (SPF) grade, were used in this study. The CCI model was established following Bennett's method^83^. After anesthesia with pentobarbital sodium (40 mg/kg), rats were placed in a prone position on the surgical table. The right hind limb was disinfected, and the sciatic nerve was exposed through a small incision. Four loose ligatures were made around the nerve using 4-0 chromic gut sutures with approximately 1 mm spacing, resulting in a constriction segment of about 4–6 mm. The ligation tightness was adjusted until a brief and slight twitch of the hind paw was observed, indicating proper nerve constriction. Muscle and skin were sutured layer by layer, and the incision site was disinfected with povidone-iodine. Postoperative care was provided under standard conditions. Successful model establishment was confirmed by signs such as reduced hind limb weight bearing, paw contracture, and excessive licking behavior. Rats were randomly divided into six groups (n = 8 per group): Control group: sciatic nerve exposed without ligation, followed by immediate wound closure; CCI group: rats underwent CCI surgery without any treatment; US-CCI group: rats received daily ultrasound stimulation (1 MHz, 0.5 W/cm², 20 min/day) after CCI surgery; Gel-CCI group: 100 μL of GelMA hydrogel was injected around the injured sciatic nerve post-CCI and crosslinked using ultraviolet light;10 mg/mL sPLLA-Gel-CCI group: 100 μL of 10 mg/mL sPLLA-Gel hydrogel was injected around the injured nerve post-CCI, followed by UV crosslinking;US-10 mg/mL sPLLA-Gel group: in addition to treatment in the sPLLA-Gel-CCI group, rats received daily ultrasound stimulation (1 MHz, 0.5 W/cm², 20 min/day). Four weeks post-surgery, all animals were euthanized, and bilateral gastrocnemius muscles, sciatic nerves, and spinal cords were harvested for subsequent analyses.

### Behavioral assessments in rats

Behavioral tests were performed on days 0, 1, 7, 14, 21, and 28 after CCI surgery to evaluate mechanical, thermal, and cold allodynia, as well as motor function recovery. Mechanical sensitivity was assessed using von Frey filaments applied perpendicularly to the plantar surface of the hind paw, and the paw withdrawal mechanical threshold (PWMT) was determined by averaging valid responses after six trials. Thermal nociception was evaluated with a hot plate maintained at 50 ± 1 °C, recording the latency to withdrawal or licking of the hind paw, with a 30 s cut-off to prevent tissue damage. Cold allodynia was tested by applying 100 μL acetone to the plantar surface, and behavioral responses within 40 s were scored on a standardized 0–3 scale. For each test, six trials were conducted per rat, with the highest and lowest values excluded, and the mean of the remaining four used for analysis. On day 28, gait analysis was performed using the CatWalk XT 10.6 system (Noldus); uninterrupted runs were recorded, and parameters including the SFI and MCMI were calculated to quantify motor coordination and functional recovery.

### Electrophysiological assessment

Electrophysiological evaluation of sciatic nerve function was performed using a biological signal acquisition and analysis system (BL-420F, Mengtai, China) with hook electrodes for both stimulation and recording of CMAP. After anesthesia, rats were placed in the prone position and the sciatic nerve was exposed. A hook-shaped stimulating electrode was placed around the nerve with an inter-electrode spacing of 1 cm. The recording electrode was positioned on the gastrocnemius muscle of the corresponding hind limb. Rectangular electrical pulses (2.5 V, 0.2 ms duration) were applied using a stimulator. The latency (Lm) and amplitude (Am) of the elicited M-wave were recorded to assess CMAP characteristics.

### WB analysis

Rats were euthanized, and the L4-L6 spinal cord segments were quickly extracted and snap-frozen in liquid nitrogen. Subsequently, tissue samples were homogenized on ice in 500 μl of RIPA lysis buffer, incubated for 30 min, and centrifuged at 12,000 rpm for 5 min (4 °C). The supernatant was collected, and protein concentration was quantified using a BCA Protein Assay Kit (Solarbio, China). SDS-PAGE electrophoresis was performed using 10% or 12.5% gels, with 20 μg of protein loaded per well. Electrophoresis was run at 80-120 V until the bromophenol blue marker reached the bottom of the separating gel. The proteins were then transferred to PVDF membranes (0.22 μm, Millipore, USA) using a constant current of 200 mA. The membranes were blocked with 5% non-fat milk at room temperature for 1 hour, followed by overnight incubation at 4 °C with primary antibodies. The following primary antibodies were used: GFAP (1:1000, CST, USA), Iba-1 (1:1000, Abcam, UK), TNF-α (1:1000, Proteintech, USA), IL-6 (1:1000, Proteintech, USA), TRPV1 (1:1000, Abcam, UK), p65 (1:5000, Proteintech, USA), β-actin (1:10000, Proteintech, USA), GAPDH (1:50000, Proteintech, USA), H-3 (1:2000, Proteintech, USA). After washing three times with TBST, HRP-conjugated secondary antibodies (1:5000, Proteintech, USA) were incubated at room temperature for 1 hour. The protein bands were visualized using a chemiluminescence detection system, and the band intensity was analyzed using ImageJ software.

### Histological staining

#### H&E staining

Tissues were fixed in 4% PFA for 72 hours, followed by graded dehydration and paraffin embedding. Sections of 5–8 μm thickness were prepared. Samples were stained using a commercial H&E staining kit (Solarbio, China). After deparaffinization with xylene and rehydration through graded ethanol, sections were stained with hematoxylin for 2 minutes, differentiated with 1% acid alcohol for 3 seconds, counterstained with eosin for 45 seconds, dehydrated again with ethanol, cleared with xylene, and mounted with neutral resin. Images were acquired using a digital pathology scanner (KFBIO, China).

#### LFB staining

After dissection, rat sciatic nerves were fixed in 4% PFA for 72 hours. Samples were embedded and sectioned as described above. Sections were deparaffinized with xylene, rehydrated to 95% ethanol, and rinsed in distilled water. Slides were incubated in Luxol Fast Blue solution (Solarbio, China) overnight at room temperature. The next day, slides were washed in 95% ethanol, differentiated in lithium carbonate solution (Solarbio, China) for 2–3 seconds, rinsed thoroughly in distilled water, dehydrated in graded ethanol, cleared in xylene, and mounted with neutral resin. Images were acquired using a digital pathology scanner (KFBIO, China).

#### Oil red O staining

Bilateral gastrocnemius muscles were collected, fixed, embedded, and sectioned. Sections were rinsed in 60% isopropanol for 20 minutes, stained with Oil Red O solution (Solarbio, China) for 15 minutes, then differentiated in 60% isopropanol until the background was clear. After thorough rinsing in distilled water, nuclei were counterstained with hematoxylin for 2 minutes, washed, and mounted using glycerol gelatin. Images were acquired using a digital pathology scanner (KFBIO, China).

#### Immunohistochemistry (IHC)

After fixation in 4% PFA, tissues were dehydrated, paraffin-embedded, and sectioned at 5 μm thickness. Sections were deparaffinized, rehydrated, and subjected to antigen retrieval in citrate buffer (pH 6.0) at 95 °C for 20 minutes. Endogenous peroxidase activity was blocked with 3% hydrogen peroxide for 15 minutes at room temperature. Non-specific binding was blocked with 5% bovine serum albumin (BSA) for 30 minutes. Sections were incubated overnight at 4 °C with primary antibodies against TRPV1 (1:100, Abcam, UK) and p65 (1:200, Proteintech, USA). After PBS washes, sections were incubated with HRP-conjugated secondary antibodies (1:500, ORIGENE, China) for 1 hour at room temperature. Color development was performed using a DAB kit (Solarbio, China), followed by hematoxylin counterstaining, dehydration, and mounting. Images were acquired using a digital pathology scanner (KFBIO, China).

#### Immunofluorescence (IF) staining

Tissues were fixed in 4% PFA, dehydrated, embedded, and sectioned. Sections were treated with 3% hydrogen peroxide for 15 minutes, followed by antigen retrieval in sodium citrate buffer (95 °C) for 20 minutes. After washing with PBS (3 times, 5 minutes each), sections were blocked with 5% BSA for 1 hour and incubated overnight at 4 °C with the following primary antibodies: GFAP (1:200, CST, USA), Iba-1 (1:200, Abcam, UK), TUJ1 (1:1000, Abcam, UK), MBP (1:200, Abcam, UK), TRPV1 (1:100, Abcam, UK), and p65 (1:200, Proteintech, USA). The next day, slides were incubated with fluorescent secondary antibodies at room temperature for 2 hours in the dark, washed with PBS, and counterstained with DAPI (Beyotime, China). After mounting with anti-fade reagent (Beyotime, China), sections were visualized and imaged using a confocal laser scanning microscope (IXplore IX85 Spin, Olympus, Japan). Fluorescence intensity was analyzed using ImageJ software.

### TEM imaging

Following dissection, rat sciatic nerves were immediately fixed in pre-cooled 2.5% glutaraldehyde for at least 24 hours. Samples were then post-fixed in 1% osmium tetroxide for 2 hours at 4 °C, dehydrated through a graded ethanol series, and embedded in resin. Ultrathin sections (approximately 100 nm) were prepared using an ultramicrotome and observed using a transmission electron microscope (Hitachi 7500). ImageJ software was used to quantify the average diameter of myelinated axons and the myelin sheath area.

### ELISA test

To quantify levels of IL-1β and PGE2 in rat spinal cord tissue, the L4–L6 segments were homogenized in 1 mL of pre-chilled RIPA lysis buffer (Beyotime, China) and incubated on ice for 30 minutes. The homogenate was centrifuged at 12,000×g for 15 minutes at 4 °C, and the supernatant was collected for ELISA assays using commercial kits (Macklin, China) to determine protein concentrations.

### RNA transcriptome sequencing

To investigate the underlying mechanisms by which 10 mg/mL sPLLA-Gel hydrogel combined with ultrasound activation promotes nerve injury repair, spinal cord tissues (L4–L6 segments) from three rats in the CCI group and three rats in the US–10 mg/mL sPLLA-CCI group were randomly selected for RNA-seq. Total RNA was extracted using the TRIzol method, and RNA quantity, purity, and integrity were assessed. Polyadenylated mRNA was enriched using oligo(dT) magnetic beads, fragmented, and used to synthesize first- and second-strand cDNA. The purified cDNA underwent end-repair, A-tailing, adaptor ligation, UDG digestion, and PCR amplification to construct strand-specific libraries (insert size: 300 ± 50 bp). Libraries were sequenced on an Illumina NovaSeq™ 6000 platform using paired-end sequencing. Raw reads were filtered to obtain high-quality data, which were aligned to the reference rat genome. Gene expression quantification, differential expression analysis, GO enrichment, and KEGG pathway analyses were performed.

### Statistical analysis

All statistical analyses were performed using GraphPad Prism 8.0.2. Data were analyzed using one-way analysis of variance (ANOVA) and t-tests, and results were expressed as mean ± standard deviation (SD). A p-value < 0.05 was considered statistically significant. All quantitative experiments were independently repeated at least three times.

## Conclusion

In summary, we have successfully developed an ultrasound-responsive, biodegradable piezoelectric hydrogel (sPLLA-Gel). This hydrogel exhibits excellent mechanical properties, injectability, and biodegradability, and is capable of generating bioelectric signals beneficial for nerve regeneration upon ultrasound activation. The sPLLA-Gel hydrogel significantly promotes neural repair and alleviates NP. Mechanistically, the hydrogel facilitates the differentiation of NSCs toward a neuronal lineage and induces the polarization of macrophages from the pro-inflammatory M1 phenotype to the anti-inflammatory M2 phenotype. It improves both behavioral and electrophysiological outcomes associated with nerve injury and effectively inhibits spinal glial cell activation and pro-inflammatory cytokine expression. Furthermore, it exerts its analgesic and neuroprotective effects by suppressing the activation of TRPV1 channels and the NF-κB signaling pathway, thereby mitigating central sensitization and inflammation-associated NP. Notably, a single injection of the hydrogel, combined with brief ultrasound stimulation, was sufficient to produce sustained therapeutic effects at the site of nerve injury. This approach significantly accelerated functional nerve recovery and provided long-lasting pain relief with excellent biocompatibility. Altogether, the ultrasound-responsive, biodegradable sPLLA-Gel hydrogel offers an innovative and noninvasive therapeutic strategy for peripheral nerve regeneration and NP management. It represents a promising and safe option for future clinical applications and opens new possibilities for the treatment of nerve injuries and pain-related disorders.

## Supplementary Material

Supplementary figures and table.

## Abbreviations

ANOVA: one-way analysis of variance
BSA: bovine serum albumin
CCI: chronic constriction injury
CMAP: compound muscle action potentials
DAPI: 4′,6-diamidino-2-phenylindole
DEGs: differentially expressed genes
DMEM: Dulbecco’s Modified Eagle Medium
DRG: dorsal root ganglion
DSC: differential scanning calorimetry
EBM: electron beam melting
ES: electrical stimulation
FDA: food and drug administration
GelMA: methacrylated gelatin
GO: Gene Ontology
H&E: hematoxylin and eosin
IHC: immunohistochemistry
IF: immunofluorescence
IFN-γ: interferon-γ
KEGG: Kyoto Encyclopedia of Genes and Genomes
LFB: Luxol Fast Blue
LH: left hind
LPS: lipopolysaccharide
MBP: myelin basic protein
MCMI: maximum contact max intensity
NP: neuropathic pain
NSAIDs: nonsteroidal anti-inflammatory drugs
NSC: neural stem cell
PFA: paraformaldehyde
PI: propidium iodide
PLLA: poly-L-lactic acid
PNI: peripheral nerve injury
PVDF: polyvinylidene fluoride
PWMT: paw withdrawal mechanical threshold
PZT: lead zirconate titanate
RGMW: relative gastrocnemius muscle weight
RNA-seq: RNA sequencing
SD: standard deviation
SFI: sciatic functional index
sPLLA: short PLLA piezoelectric nanofibers
SPF: specific pathogen-free
TEM: transmission electron microscopy
TRP: transient receptor potential
US: ultrasound
