# Supplementary material for: The role of an ultrasound-responsive injectable piezoelectric hydrogel in promoting nerve regeneration and alleviating neuropathic pain
Source: Theranostics. 2026 Jun 25;16(14):7780–805. doi: 10.7150/thno.131276 (PMC13440276; doi:10.7150/thno.131276)
Supplement: Supplementary file 1 [file thnov16p7780s1.pdf]

Supporting Information

The role of an ultrasound-responsive injectable piezoelectric hydrogel in promoting nerve regeneration and alleviating neuropathic pain

Zhaoyang Guo[1#], Weiqian Jiang[1#], Wentao Zhang[1], Zhongju Liu[3], Hang Zhou[1], Hang Liu[1], Lin Wang[6], Jiahao Zhang[7], Xinliang Peng[1], Xingyu Yang[1], Maohui Li[1], Hanchao Liang[1], Zhongyuan He[1], Rui Deng[1], Yongjun Dang[5], Wei Fu[2], Keyu Wei[1], Chao Xie[4], Zhong-Liang Deng[1*], Youliang Ren[2*], Lei Chu[1,8*]

[1]Department of Orthopaedics, The Second Affiliated Hospital of Chongqing Medical University, Chongqing 400010, P. R. China
[2]Department of Orthopaedics, Guizhou Provincial People's Hospital, Guiyang, 550000, PR China
[3]Department of Plastic and Maxillofacial Surgery, The Second Affiliated Hospital of Chongqing Medical University, Chongqing 400010, P. R. China
[4]Center for Musculoskeletal Research, University of Rochester Medical Center, Rochester, NY 14642, USA
[5]Novel Target and Therapeutic Intervention Laboratory, The Second Affiliated Hospital of Chongqing Medical University, Chongqing 400010, P. R. China
[6]National Engineering Research Center for Tissue Restoration and Reconstruction South China University of Technology, Guangzhou, China
[7]Health Science Center, Xi'an Jiaotong University, 76 Yanta West Road, Xi'an, 710061, P. R. China
[8]Department of Pain Management, The Second Affiliated Hospital of Chongqing Medical University, Chongqing 400010, P. R. China

[#] Zhaoyang Guo, and Weiqian Jiang are co-first authors who contributed equally to this study.

[*]Corresponding author: Lei Chu, Department of Orthopaedics, Second Affiliated Hospital of Chongqing Medical University,76 Linjiang Road, Yuzhong Distinct, Chongqing, 400010, P. R. China or 302939@hospital.cqmu.edu.cn；
Youliang Ren, Department of Orthopaedics, Guizhou Provincial People's Hospital, Guiyang, 550000, PR China or Youliang_Ren@163.com;
Zhong-Liang Deng, Department of Orthopaedics, Second Affiliated Hospital of Chongqing Medical University,76 Linjiang Road, Yuzhong Distinct, Chongqing, 400010, P. R. China or 300443@hospital.cqmu.edu.cn

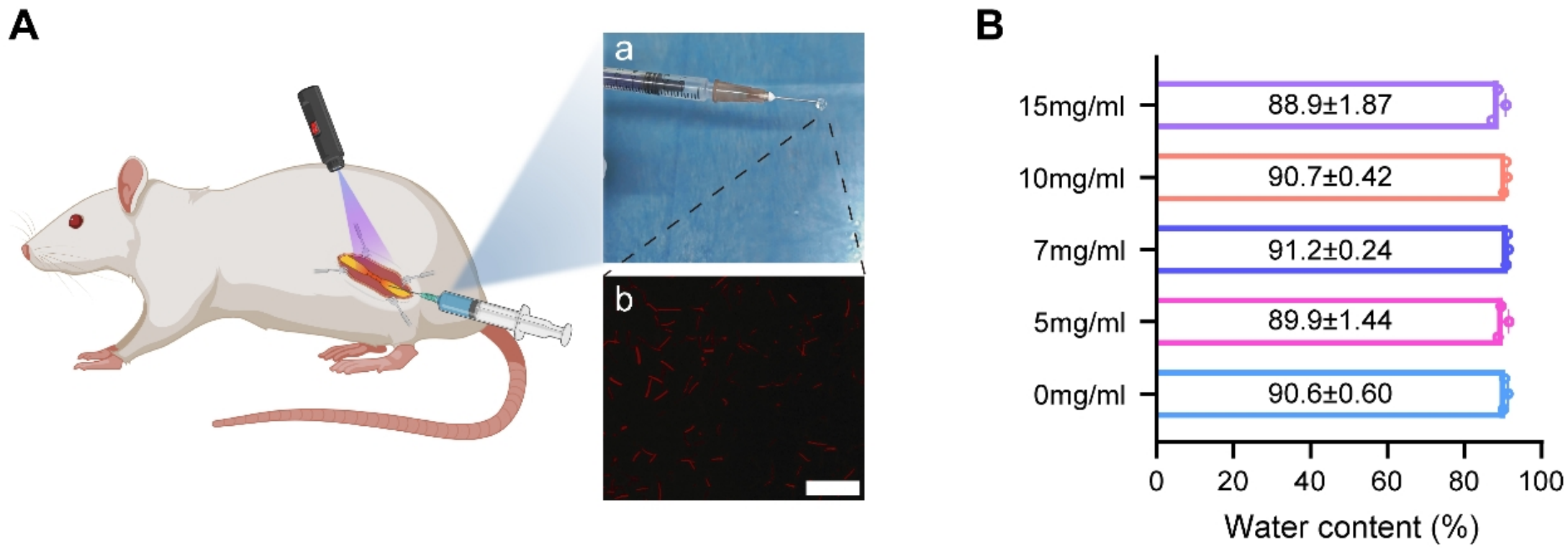

Figure S1. Characterization of the injectability and water content of GelMA and 10 mg/mL sPLLA-Gel hydrogels.(A) Schematic illustration of using piezoelectric hydrogel for peripheral nerve injury treatment. (a) Injectability test of the piezoelectric hydrogel. (b) Fluorescence image of Rhodamine B-stained sPLLA (red) (scale bar: 200 μm). (B) Water content of sPLLA-Gel hydrogels with different sPLLA concentrations.

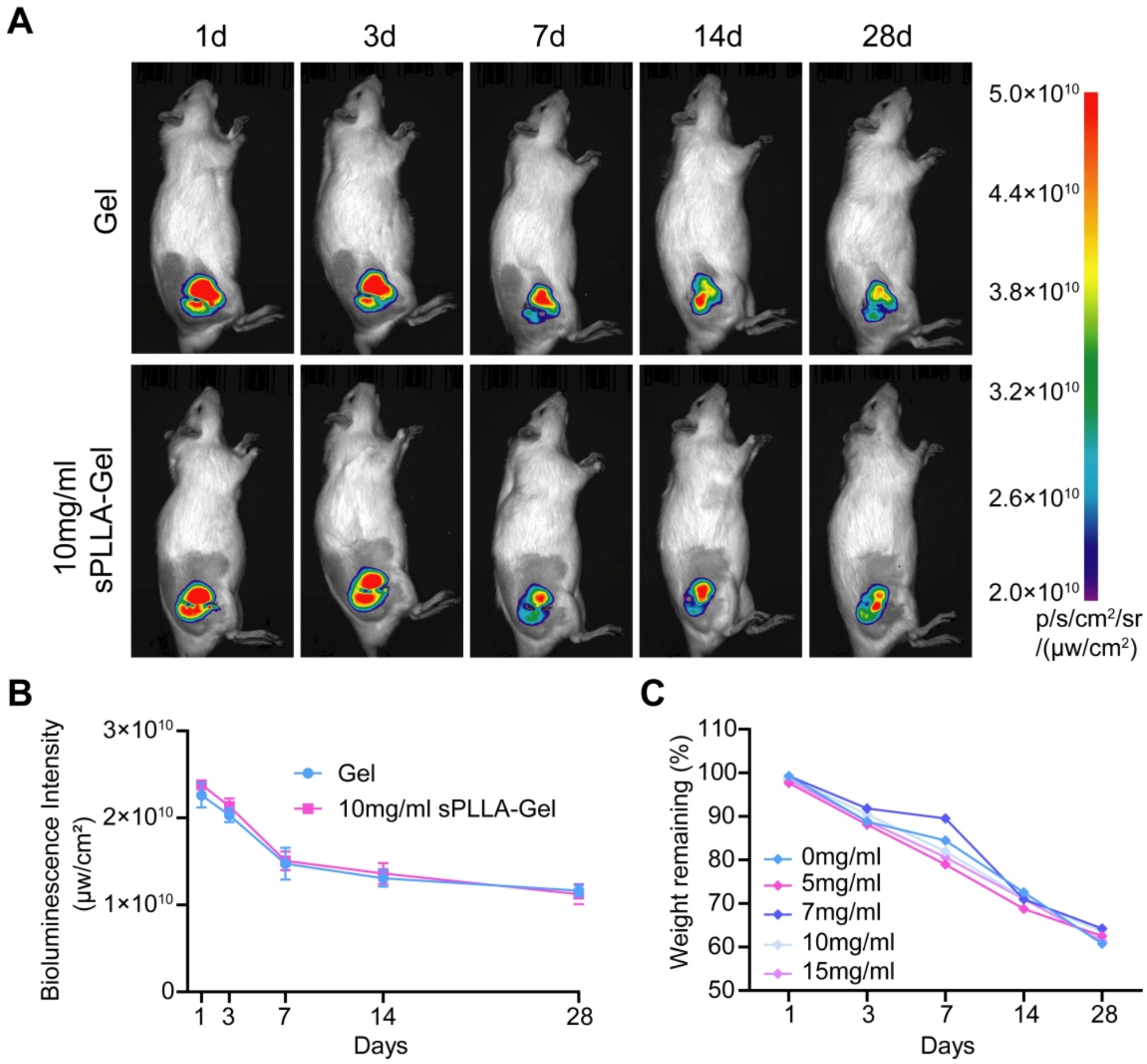

Figure S2. In vivo and in vitro degradation of GelMA and 10 mg/mL sPLLA-Gel hydrogels.(A) In vivo degradation imaging of GelMA and 10 mg/mL sPLLA-Gel in rats. (B) ROI analysis of bioluminescent signals showing no significant difference in the degradation rate and ratio between GelMA and sPLLA-Gel in vivo (n = 3). (C) Degradation profiles of sPLLA-Gel hydrogels with different sPLLA concentrations in PBS buffer containing 1 mg/mL lysozyme at 37 °C (n = 3).

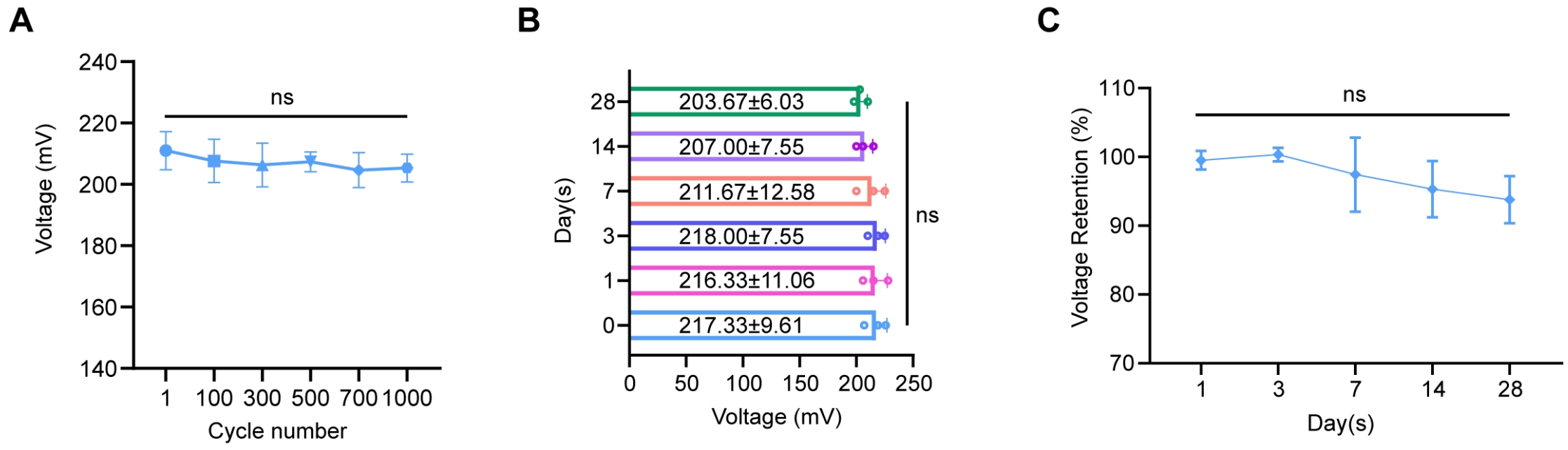

Figure S3. Stability of piezoelectric output of sPLLA-Gel under cyclic ultrasound stimulation and during in vitro degradation. (A) Cyclic stability of piezoelectric output of sPLLA-Gel under repeated ultrasound stimulation. (B) Output voltage of 10 mg/mL sPLLA-Gel hydrogel measured after lyophilization at different time points during in vitro degradation (n = 3). (C) Normalized voltage retention at each time point relative to day 0 (n = 3).

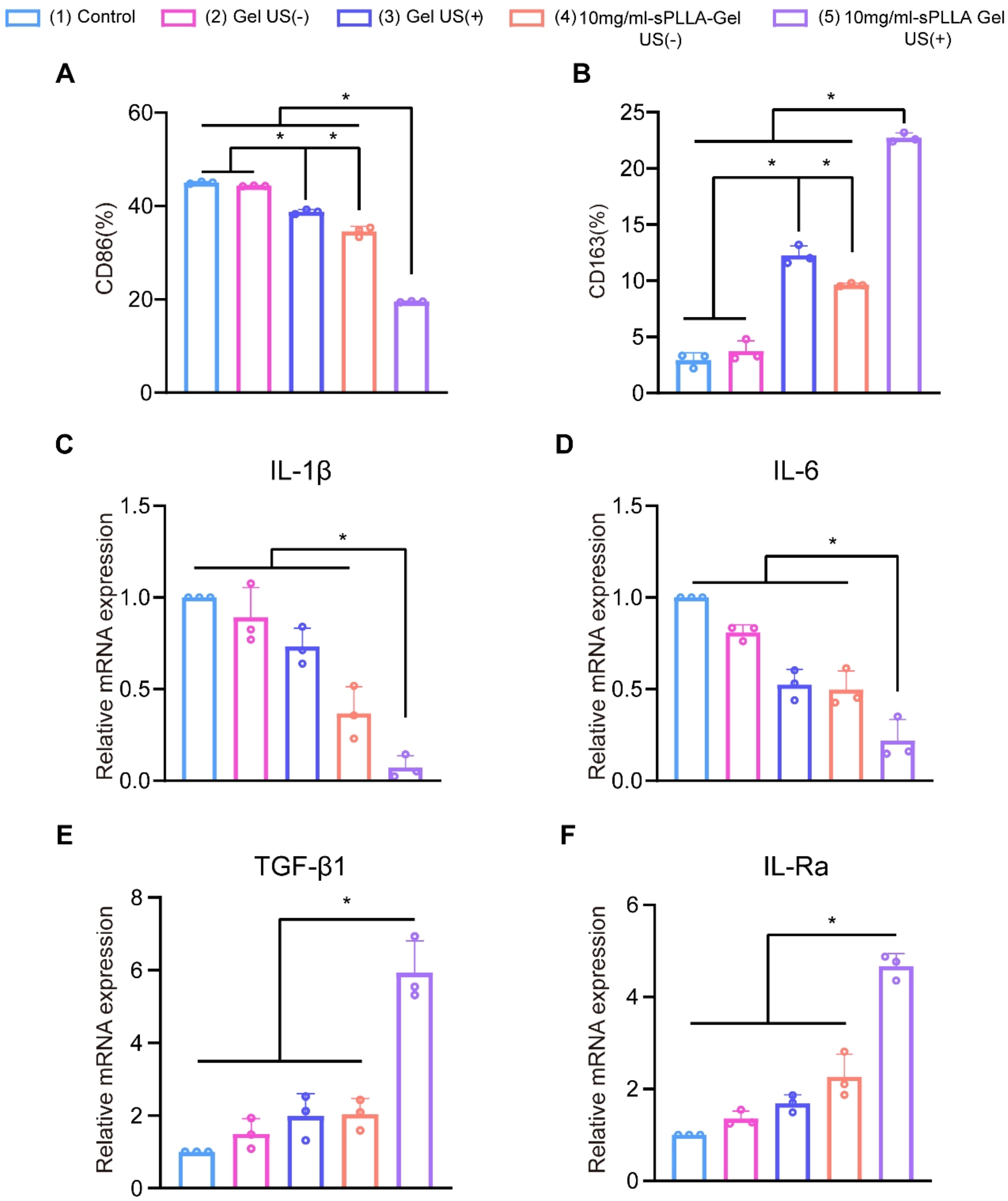

Figure S4. Quantitative analysis of FCM results for (A) CD86 and (B) CD163, and RT-qPCR detection of pro-inflammatory (IL-6 and IL-1β) (C, D) and anti-inflammatory (TGF-β1 and IL-1Ra) (E, F) gene expression in macrophages (n = 3). $P < 0.05$.

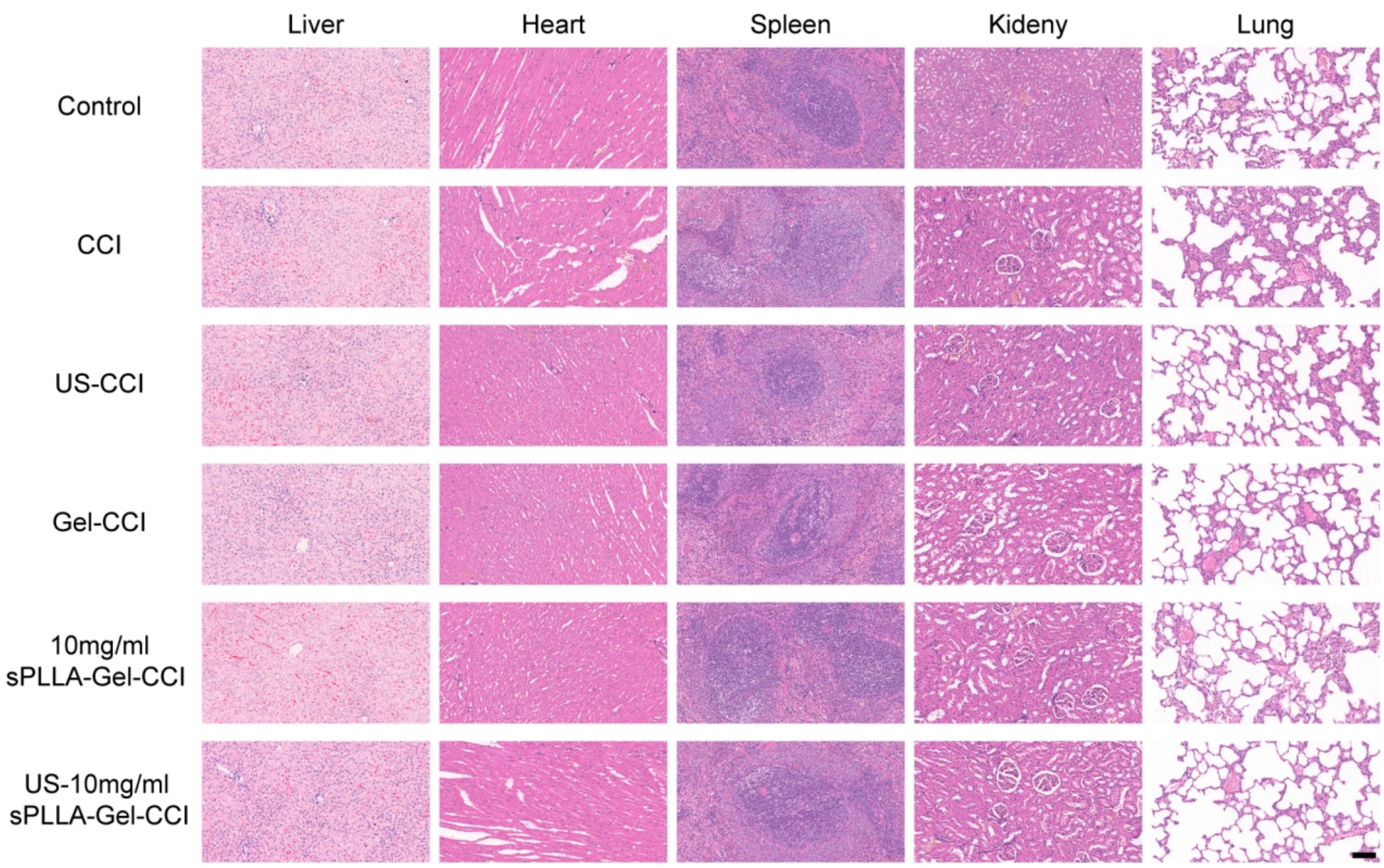

Figure S5. HE staining of major organs from rats in each group (scale bar: 100 μm).

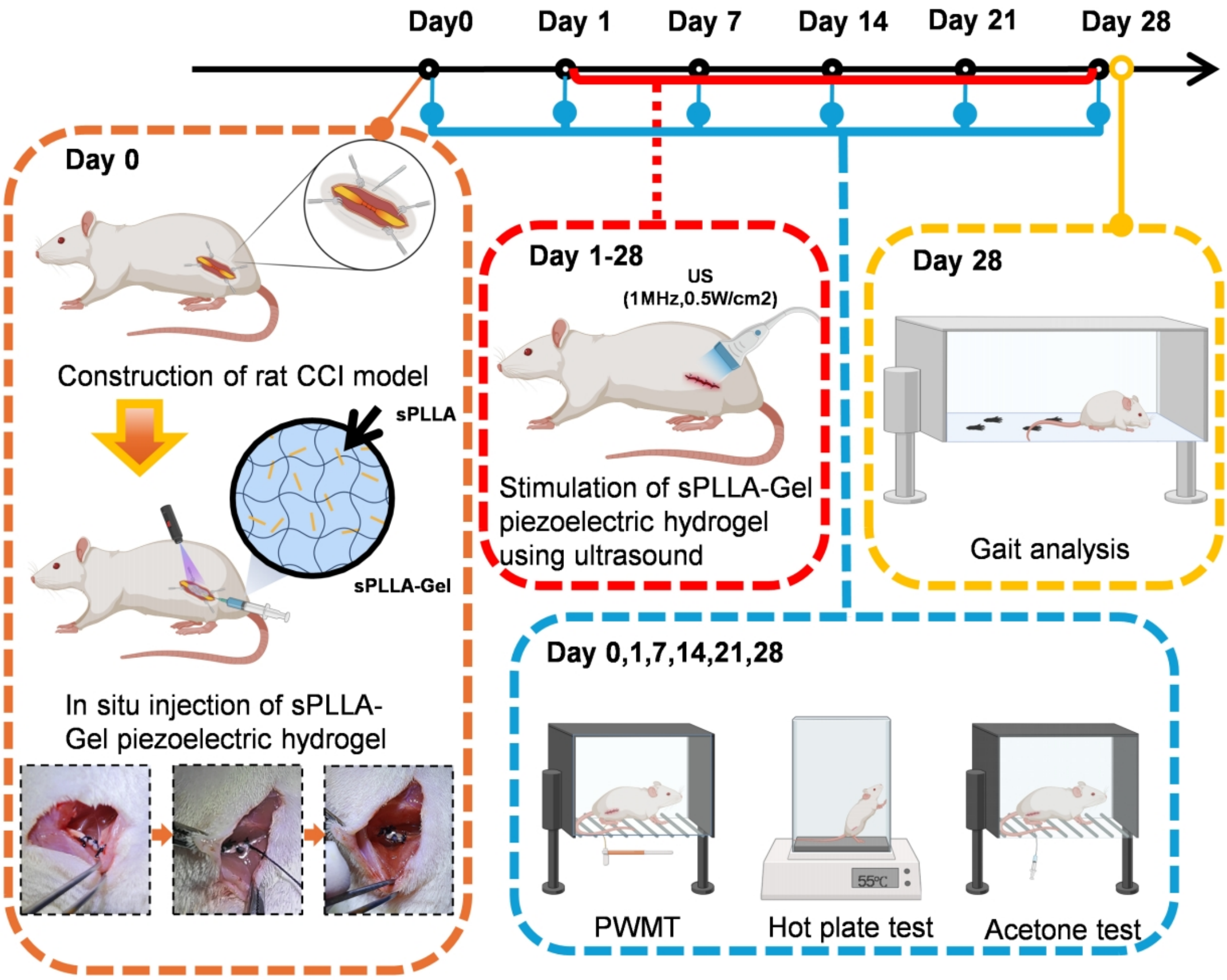

Figure S6. Schematic timeline of in vivo experiments and behavioral assessments.

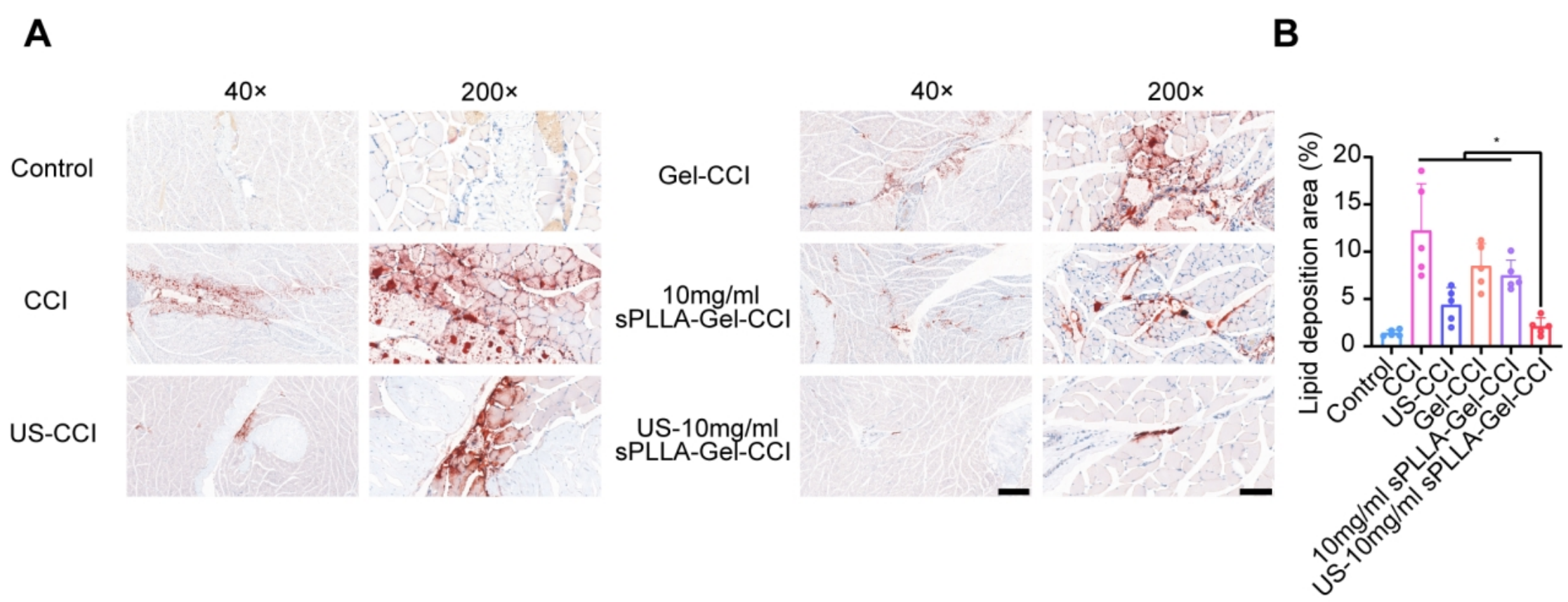

Figure S7. Oil Red O staining of the gastrocnemius muscle in rats from each group at 28 days post-surgery (A) and semi-quantitative analysis of lipid droplet area ratio (B) .(40×, scale bar: 500 μm; 200×, scale bar: 100 μm) (n = 5).

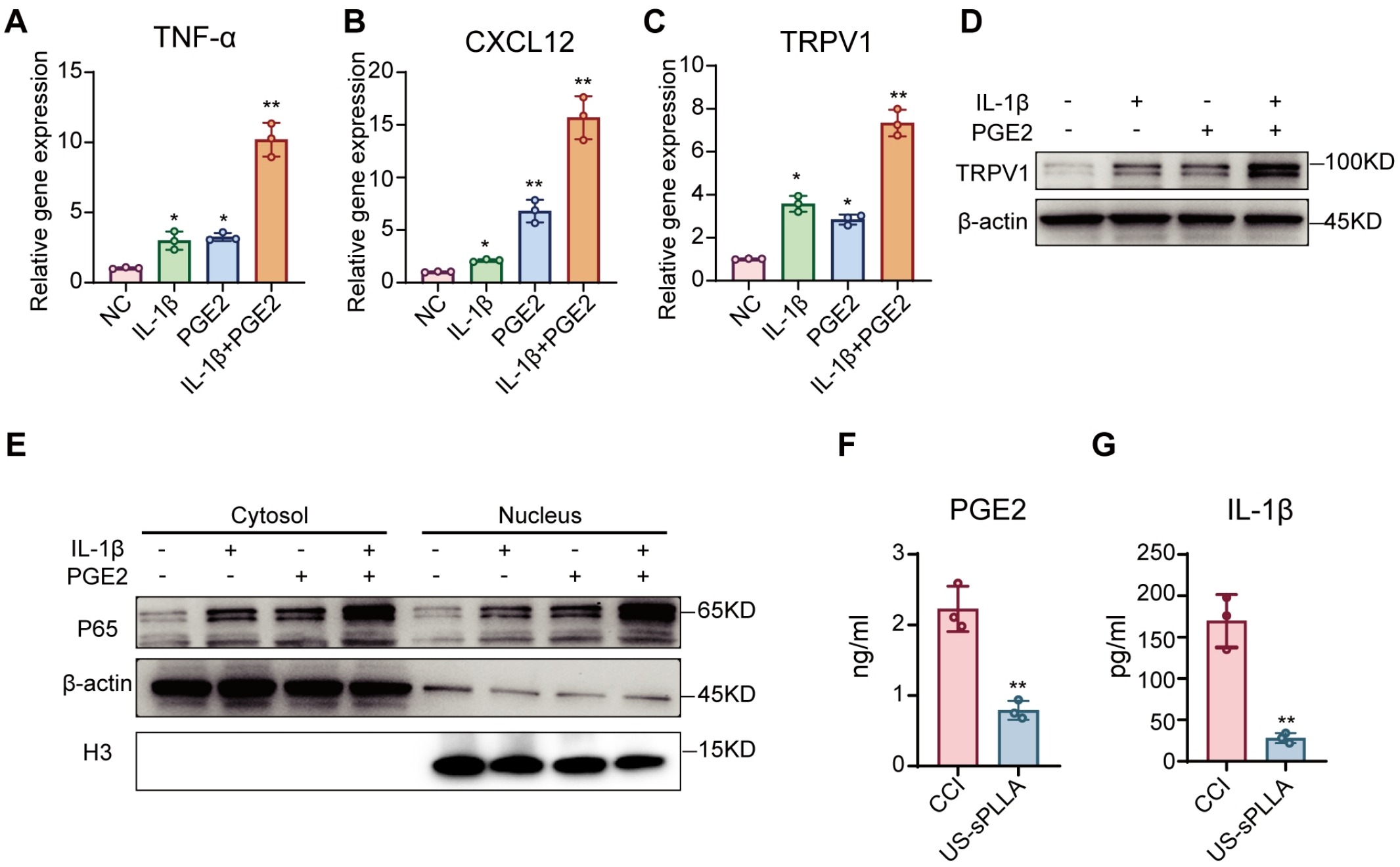

Figure S8. (A–C) Statistical results of qRT-PCR after 7 days of induction of BV2 cells with IL-1β and PGE2 (n = 3). (D) Western blot results after 7 days of induction with IL-1β and PGE2 showing that both IL-1β and PGE2 activated TRPV1 channel protein. (E) Nuclear and cytoplasmic fractionation Western blot results of BV2 cells treated with IL-1β and PGE2 demonstrating that both factors activated p65 protein and induced NF-κB nuclear translocation. (F–G) ELISA analysis of spinal cord tissues from the CCI group and the US-sPLLA group showing that the US-sPLLA hydrogel effectively reduced the production of PGE2 and IL-1β (n = 3).

Supplementary Tables

Table S1. Primers used for RT-qPCR analysis.

Gene	Forward primer (5’-3’)	Reverse primer (5’-3’)
IL-6	CTTGGGACTGATGCTGGTGA	TTGGGAGTGGTATCCTCTGTGA
IL-1β	TGGAGAGTGTGGATCCCAAG	GGTGCTGATGTACCAGTTGG
TGF-β1	CAGTACAGCAAGGTCCTTGC	ACGTAGTAGACGATGGGCAG
IL1Ra	CTCCAGCTGGAGGAAGTTAAC	CTGACTCAAAGCTGGTGGTG
TNF-α	AGGCACTCCCCCAAAAGATG	CTGGAAGACTCCTCCCAGGT
CXCL12	CGGTAAACCAGTCAGCCTGA	TCACATCTTGAGCCTCTTGTT
TRPV1	AGCCCCACATCTTTGCTACC	GTGTAATGCTGTCTGGCCCT
